# Transcriptional profiling reveals molecular signatures associated with HIV permissiveness in Th1Th17 cells and identifies *Peroxisome Proliferator-Activated Receptor Gamma* as an intrinsic negative regulator of viral replication

**DOI:** 10.1186/1742-4690-10-160

**Published:** 2013-12-21

**Authors:** Annie Bernier, Aurélie Cleret-Buhot, Yuwei Zhang, Jean-Philippe Goulet, Patricia Monteiro, Annie Gosselin, Sandrina DaFonseca, Vanessa Sue Wacleche, Mohammad-Ali Jenabian, Jean-Pierre Routy, Cécile Tremblay, Petronela Ancuta

**Affiliations:** 1 Department of Microbiology, Infectiology, and Immunology, Université de Montréal, Faculty of Medicine, Montreal Quebec, Canada; 2 CHUM-Research Centre, 900 rue Saint-Denis, Tour Viger, room R09.416, Montréal, Quebec H2X 0A9, Canada; 3 Faculty of Medicine, CARTaGENE, Université de Montréal, Montreal Quebec, Canada; 4 Department of Pediatrics, Faculty of Medicine, Ste Justine Hospital Research Center, Université de Montréal, Montreal Quebec, Canada; 5 Chronic Viral Illness Service, McGill University Health Centre, Montreal Quebec, Canada; 6 Research Institute, McGill University Health Centre, Montreal Quebec, Canada; 7 Division of Hematology, McGill University Health Centre, Montreal Quebec, Canada

**Keywords:** HIV, CD4^+^ T-cells, Th1Th17, Th1, cDNA microarrays, PPARγ

## Abstract

**Background:**

We previously demonstrated that primary Th1Th17 cells are highly permissive to HIV-1, whereas Th1 cells are relatively resistant. Molecular mechanisms underlying these differences remain unknown.

**Results:**

Exposure to replication competent and single-round VSV-G pseudotyped HIV strains provide evidence that superior HIV replication in Th1Th17 *vs.* Th1 cells was regulated by mechanisms located at entry and post-entry levels. Genome-wide transcriptional profiling identified transcripts upregulated (n = 264) and downregulated (n = 235) in Th1Th17 *vs.* Th1 cells (p-value < 0.05; fold change cut-off 1.3). *Gene Set Enrichment Analysis* revealed pathways enriched in Th1Th17 (nuclear receptors, trafficking, p38/MAPK, NF-κB, p53/Ras, IL-23) *vs.* Th1 cells (proteasome, interferon α/β). Differentially expressed genes were classified into biological categories using *Gene Ontology*. Th1Th17 cells expressed typical Th17 markers (IL-17A/F, IL-22, CCL20, RORC, IL-26, IL-23R, CCR6) and transcripts functionally linked to regulating cell trafficking (CEACAM1, MCAM), activation (CD28, CD40LG, TNFSF13B, TNFSF25, PTPN13, MAP3K4, LTB, CTSH), transcription (PPARγ, RUNX1, ATF5, ARNTL), apoptosis (FASLG), and HIV infection (CXCR6, FURIN). Differential expression of CXCR6, PPARγ, ARNTL, PTPN13, MAP3K4, CTSH, SERPINB6, PTK2, and ISG20 was validated by RT-PCR, flow cytometry and/or confocal microscopy. The nuclear receptor PPARγ was preferentially expressed by Th1Th17 cells. PPARγ RNA interference significantly increased HIV replication at levels post-entry and prior HIV-DNA integration. Finally, the activation of PPARγ pathway *via* the agonist Rosiglitazone induced the nuclear translocation of PPARγ and a robust inhibition of viral replication.

**Conclusions:**

Thus, transcriptional profiling in Th1Th17 *vs*. Th1 cells demonstrated that HIV permissiveness is associated with a superior state of cellular activation and limited antiviral properties and identified PPARγ as an intrinsic negative regulator of viral replication. Therefore, triggering PPARγ pathway *via* non-toxic agonists may contribute to limiting covert HIV replication and disease progression during antiretroviral treatment.

## Background

Despite the success of current antiretroviral therapies (ART), persistence of HIV-1 reservoirs represents a major barrier against viral eradication [1-4]. CD4^+^ T-cells are key players for antiviral immunity [5] but also main targets for productive HIV infection and long-term persistence [3,6]. The discovery of new molecular mechanisms underlying the ability of HIV to select its targets and the identification of new therapeutic strategies to block this process represent an open field of investigations in the framework of current efforts toward HIV eradication [2]. The status of HIV permissiveness in a given cell subset is dependent in part on virus ability to counteract intrinsic cellular defenses mechanisms [7] mediated by several restriction factors including APOBEC3G [8-10], TRIM5α [11,12], Tetherin/CD317 [13] and most recently discovered, SAMHD1 [14,15]. In addition to restriction factors that directly target the virus, p21*/*CDKN1A*,* a potent inhibitor of cyclin dependent kinases, was demonstrated to limit HIV replication in macrophages [16] and CD4^+^ T-cells from HIV elite controllers [17,18], likely by an indirect mechanism. On the other hand, HIV uses the host-cell machinery for its successful replication. The receptor CD4 and coreceptors CCR5 and CXCR4 were the first HIV-dependency factors (HDFs) described for HIV entry [19,20]. Several other HDFs acting at post-entry levels were identified in the last years using genome-wide RNA interference screenings in HeLa [21,22], 293 T [23] and Jurkat cell lines [24] and other high throughput techniques [25]. These studies revealed large lists of HDFs with very limited overlap when transcripts were analyzed individually [25,26]. However, when HDFs identified in each screen were functionally classified using gene ontology (GO), a greater level of overlap was observed for processes such as Nuclear pore/transport, DNA-Repair, Ubiquitin-associated/Proteasome, Mediator Complex/Transcription, RNA binding, GTP Binding, and Helicase [25]. The NF-κB, *peroxisome proliferator-activated receptor* (PPAR), and retinoic acid receptor (RAR) activation pathways were identified as being critical in two studies [21,24]. Nevertheless, some well known permissiveness factors (*e.g.,* cyclophilin A, LEDGF/p75) were not identified in these screens [25], suggesting that many other factors important for HIV permissiveness remain to be identified, especially in primary CD4^+^ T-cells.

The chemokine receptors CXCR3, CCR4, and CCR6 are markers for memory CD4^+^ T-cells subsets with distinct polarization potential, antigenic specificity, and permissiveness to HIV [27,28]. CXCR3^+^CCR4^-^CCR6^+^ T-cells exhibit a Th1Th17 polarization profile as they express transcription factors and produce cytokines specific for both Th1 (T-bet and IFN-γ) and Th17 (RORC and IL-17) lineages, while CXCR3^+^CCR4^-^CCR6^-^ T-cells express functional markers specific for the Th1 lineage only [29,30]. In addition, CXCR3^+^CCR4^-^CCR6^+^/Th1Th17 cells are specific to pathogens such as *M. tuberculosis* and *C. albicans*, while CXCR3^+^CCR4^-^CCR6^-^/Th1 cells proliferate in response to CMV [29,30]. We previously reported that: *(i)* Th1Th17 cells are highly permissive to replication-competent R5 and X4 HIV strains, while Th1 cells are relatively resistant; (ii) CCR6^+^ T-cells (including Th1Th17 cells) are major cellular targets of infection *in vivo*; and that *(iii)* the frequency of Th1Th17 but not Th1 cells is dramatically reduced in HIV-infected subjects *vs.* uninfected controls, with viral suppressive ART being inefficient in restoring Th1Th17 paucity [31]. Distinct HIV replication in Th1Th17 *vs.* Th1 cells is consistent with findings by other groups that CMV-specific but not *M. tuberculosis*-specific cells are protected from HIV infection by an autocrine production of CCR5 binding chemokines [32,33]. Differences in HIV permissiveness between Th1Th17 and Th1 cells may account for the depletion of *M. tuberculosis*-specific cells [34] and the deleterious consequences of CMV-specific cell persistence in HIV-infected subjects [35]. Although Th1Th17 and Th1 cells produce similar CCL3 and CCL5 levels, a superior CCR5 expression on Th1Th17 cells *ex vivo* argue in favor of their increased ability to support R5 HIV entry [31]. However, the expression of CXCR4 was similar on Th1Th17 and Th1 cells despite the fact that permissiveness to X4 HIV strains is restricted to Th1Th17 cells [31], suggesting the possibility that additional post-entry mechanisms likely regulate HIV replication in Th1 cells. Indeed, a very recent study demonstrated that HIV restriction in CMV-specific cells (Th1 polarized) is mediated by post-entry antiviral mechanisms linked to type-I IFN responses and the selective expression of TRIM22 and TRIM5 restriction factors [36]. Thus, understanding molecular differences between Th1Th17 and Th1 cells may provide new insights into molecular mechanisms of HIV permissiveness *vs.* restriction in primary CD4^+^ T-cell subsets and reveal new therapeutic avenues.

In this study we aimed to identify molecular mechanisms underlying differences in HIV permissiveness between HIV-permissive Th1Th17 and HIV-resistant Th1 cells. This study provides a unique genome-wide characterization of differential gene expression in Th1Th17 *vs.* Th1 cells, reveals pathways and biological functions linked to HIV permissiveness, and identifies the PPARγ activation pathway as the “*Achilles’ heel*” for HIV permissiveness that may be therapeutically targeted to limit viral replication in primary CD4^+^ T-cells.

## Results

### Superior HIV permissiveness in Th1Th17 *vs.* Th1 cells upon exposure to replication-competent and single-round viruses

**Figure 1:**
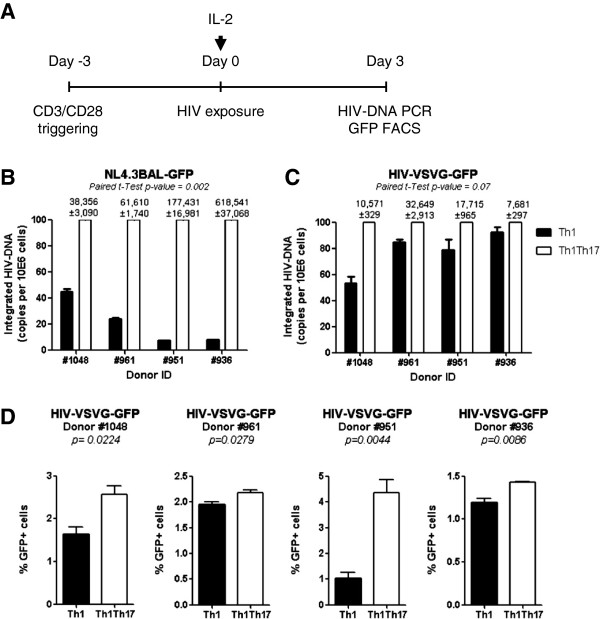
**Superior HIV replication in Th1Th17 *vs. *Th1 cells is regulated at entry and post-entry level.** Matched Th1 and Th1Th17 cells were sorted by flow cytometry from four different HIV-uninfected subjects and stimulated *via* CD3/CD28 for 3 days **(A)**. Cells were exposed to replication competent NL4.3BAL-GFP **(B)** or single round HIV-VSVG-GFP pseudotyped strains (50 ng HIV-p24/10^6^ cells) **(C-D)** for 3 h at 37°C. Unbound virus was removed by extensive washing, and cells were cultured for 3 additional days in the presence of IL-2 (5 ng/ml). Integrated HIV-DNA and GFP expression levels were quantified by nested real-time PCR **(B-C)** and flow cytometry **(D)**, respectively, at day 3 post-infection. Shown in **B-C** is relative HIV-DNA integration in Th1 *vs.* Th1Th17 cells (normalized to the maximal value considered to be 100% in Th1Th17 cells); values above graphs are integrated HIV-DNA copies *per* 10^6^ cells in Th1Th17 cells (mean±SD of triplicate wells). Paired *t*-Test values are indicated on the graphs.

The increased expression of CCR5 on Th1Th17 *vs.* Th1 cells [31] suggests a superior ability of Th1Th17 cells to support HIV entry. To distinguish between entry and post-entry regulation mechanisms, HIV integration in Th1Th17 and Th1 cells was quantified upon exposure to replication competent R5 HIV strain (NL4.3BAL-GFP) or single-round VSV-G-pseudotyped HIV (HIV-VSVG-GFP) that enters cells independently of the HIV receptor CD4 and coreceptors CCR5 and CXCR4 [37,38]. To this aim, highly pure matched Th1Th17 and Th1 subsets were sorted by flow cytometry from four different donors (Additional file 1: Figure S1), stimulated *via* CD3/CD28 for 3 days, and exposed to HIV (Figure 1A). HIV-DNA integration in Th1Th17 *vs.* Th1 cells was significantly higher when cells were exposed to NL4.3BAL-GFP (Figure 1B), similar to our previous findings [31]. Differences were marginally significant when cells were exposed to HIV-VSVG-GFP (Figure 1C). Levels of GFP-expression, an indicator of HIV transcription, were slightly higher in Th1Th17 *vs.* Th1 cells, with donor-to-donor variations being observed (Figure 1D). Because the stage of cells differentiation may be linked to HIV permissiveness, we investigated the central memory (CM, CD45RA^-^CCR7^+^) *vs.* effector memory (EM, CD45RA^-^CCR7^-^) phenotype in Th1Th17 and Th1 subsets, using previously described markers [6,39,40]. No significant differences were found between Th1Th17 and Th1 subsets in terms of CM/EM ratios (data not shown). In addition, similar differences were observed in HIV permissiveness between Th1Th17 and Th1 cells when infection was performed on sorted CM and EM subsets (data not shown). These results provide evidence that HIV permissiveness in memory Th1Th17 *vs.* Th1 cells is mainly regulated at the entry level; however, post-entry mechanisms located at the pre- and/or post-integration level likely contribute to these differences.

### Distinct gene expression profiles in Th1Th17 *vs.* Th1 cells

**Figure 2:**
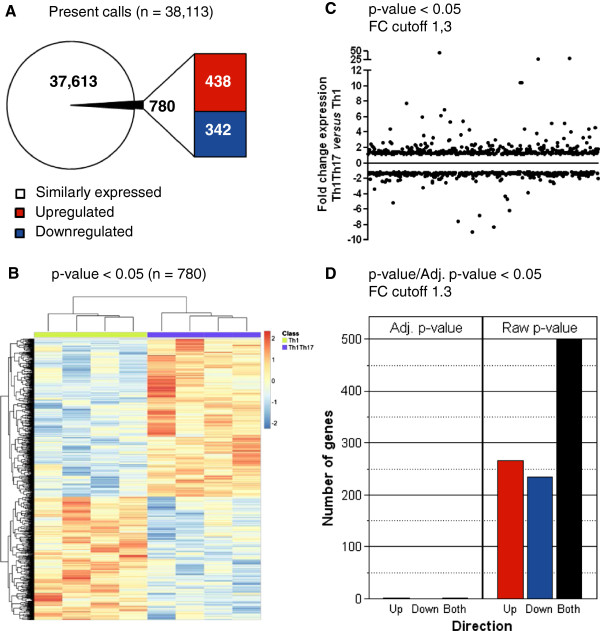
**Identification of differentially expressed genes in Th1Th17 *vs. *Th1 cells.** Matched Th1Th17 and Th1 subsets from four HIV-uninfected donors were sorted and stimulated as in Figure 1. Total RNA was extracted and reverse transcribed into cDNA that was then hybridized on the Human Genome U133 Plus 2.0 Array (Affymetrix). Statistical analysis using one-way ANOVA was performed to identify differentially expressed genes (p-value <0.05) and the differential expression fold change (FC) was calculated. **(A)** Shown is a schematic representation of the number of ”*present calls*” shared (n = 38,113) and differentially expressed (n = 438 upregulated and n = 342 downregulated) in Th1Th17 *vs.* Th1 (p-value <0.05). **(B)** Hierarchical clustering analysis of differentially expressed probe sets separated the 8 samples in two groups corresponding to Th1Th17 and Th1 subsets. **(C)** Depicted is the fold change of differentially expressed genes in Th1Th17 *vs.* Th1 (p-value <0.05 and FC cut off 1.3). **(D)** Shown are numbers of probe sets differentially expressed in Th1Th17 *vs.* Th1 with a fold change cut-off of 1.3 according to their p-value (n = 265 upregulated in red and n = 235 downregulated in blue) or adjusted p-value (n = 2 upregulated).

To identify transcriptional signatures associated with HIV permissiveness and resistance in Th1Th17 and Th1 cells, respectively, we used the Affymetrix technology (Gene Chip® Human Genome U133 Plus 2.0 Array) for a genome-wide transcriptional analysis in matched Th1Th17 and Th1 cells isolated from the peripheral blood of four uninfected individuals and stimulated *via* CD3/CD28 for 3 days *in vitro*. The choice of this timing is justified by the fact that robust differences in HIV permissiveness between Th1Th17 *vs.* Th1 cells were observed in our previous studies when cells were exposed to the virus at day 3 post TCR triggering [31]. The primary analysis revealed 38,113 present calls, with 780 probe sets that were differentially expressed in Th1Th17 *vs.* Th1 cells (one-way ANOVA p-value <0.05): 438 probe sets upregulated (corresponding to 392 known genes and 46 unknown transcripts) and 342 downregulated (corresponding to 268 known genes and 74 unknown transcripts) (Figure 2A-B; Additional file 2: Table S1 and Additional file 3: Table S2). Further, 265 and 235 probe sets were upregulated and downregulated, respectively, in Th1Th17 *vs.* Th1 subsets with a fold change (FC) superior to 1.3 (Figure 2C-D; Additional file 2: Table S1 and Additional file 3: Table S2). Transcripts upregulated in Th1Th17 *vs.* Th1 cells (p-value <0.05, FC cut-off 1.3) include known markers of Th17 cells such as IL-17A, IL-22, CCL20, IL-17 F, RORC, IL-26, IL-23R, CCR6, IL1R1, and CSF2/GM-CSF (Table 1). When the adjusted p-value (adj. p-value <0.05) was calculated, two Th17-specific genes were identified as being highly expressed in Th1Th17 *vs.* Th1 subsets: the transcription factor RORC and the cytokine IL-22 (Additional file 2: Table S1). These findings provide a first validation of the transcriptional results obtained by microarray studies. In addition, the analysis of top differentially expressed genes reveals new markers for Th1Th17 cells including CTSH, PTPN13, CXCR6, MCAM, CCR2, PPARγ, TNFSF13B, ARNTL, FURIN, MAP3K4, and CEACAM1 (Table 1) and for Th1 cells including CXCL10, PTK2, CXCR5, PECAM1, CCL17, ALCAM, and GRK5 (Table 2). Thus, Th1Th17 and Th1 cells distinguish from each other by a set of transcripts (corresponding to 660 known genes and 120 unknown transcripts) that may be involved in the differential regulation of HIV permissiveness *vs.* restriction in these cells.

**Table 1:** **Selected genes upregulated in Th1Th17 *vs. *** **Th1**

**Gene symbol**	**Fold change Th1Th17/Th1**	**p-value**	**Gene title**
IL17A	44,94	0,000	Interleukin 17A
IL22	29,18	0,000	Interleukin 22
CCL20	26,69	0,000	Chemokine (C-C motif) ligand 20
IL17F	10,47	0,039	Interleukin 17 F
RORC	7,73	0,000	RAR-related orphan receptor C
IL26	6,90	0,000	Interleukin 26
IL23R	6,14	0,000	Interleukin 23 receptor
CTSH	5,46	0,000	Cathepsin H
CCR6	5,38	0,001	Chemokine (C-C motif) receptor 6
HPGD	5,16	0,004	Hydroxyprostaglandin dehydrogenase 15-(NAD)
PTPN13	4,45	0,000	Protein tyrosine phosphatase, non-receptor type 13
KLRB1	3,87	0,040	Killer cell lectin-like receptor subfamily B, member 1
IL1R1	3,39	0,004	Interleukin 1 receptor, type I
CSF2	2,68	0,041	Colony stimulating factor 2 (granulocyte-macrophage)
LST1	2,60	0,001	Leukocyte specific transcript 1
CXCR6	2,44	0,003	Chemokine (C-X-C motif) receptor 6
MCAM	2,27	0,001	Melanoma cell adhesion molecule
CCR2	2,25	0,034	Chemokine (C-C motif) receptor 2
RORA	2,14	0,005	RAR-related orphan receptor A
CD80	2,08	0,013	CD80 molecule
IL18RAP	2,05	0,049	Interleukin 18 receptor accessory protein
PPARG	2,04	0,019	Peroxisome proliferator-activated receptor gamma
TNFRSF25	2,01	0,008	Tumor necrosis factor receptor superfamily, member 25
TNFSF13B	1,95	0,014	Tumor necrosis factor (ligand) superfamily, member 13b
LTB	1,87	0,012	Lymphotoxin beta (TNF superfamily, member 3)
ARNTL	1,72	0,024	Aryl hydrocarbon receptor nuclear translocator-like
IL15	1,69	0,012	Interleukin 15
FURIN	1,61	0,040	Furin (paired basic amino acid cleaving enzyme)
IL12RB1	1,56	0,031	Interleukin 12 receptor, beta 1
ATF5	1,54	0,004	Activating transcription factor 5
THY1	1,54	0,043	Thy-1 cell surface antigen
MAP3K4	1,53	0,003	Mitogen-activated protein kinase kinase kinase 4
CEACAM1	1,52	0,021	Carcinoembryonic antigen-related cell adhesion molecule 1
MAP3K4	1,52	0,006	Mitogen-activated protein kinase kinase kinase 4
IL2	1,51	0,041	Interleukin 2
ATF5	1,46	0,018	Activating transcription factor 5
CTSC	1,43	0,023	Cathepsin C
CD40LG	1,45	0,037	CD40 ligand
RUNX1	1,40	0,012	Runt-related transcription factor 1
PTEN	1.39	0.031	Phosphatase and tensin homolog
CD28	1,37	0,046	CD28 molecule
TRIM8	1,36	0,011	Tripartite motif-containing 8
CASP4	1,34	0,025	Caspase 4, apoptosis-related cysteine peptidase
CYP27B1	1,33	0,031	Cytochrome P450, family 27, subfamily B, polypeptide 1
FASLG	1,33	0,046	Fas ligand (TNF superfamily, member 6)

Shown are 44 differentially expressed genes (p-value <0.05 and cut-off 1.3 fold) that are preferentially expressed in Th1Th17 *vs*. Th1 cells.

**Table 2:** **Selected genes downregulated in Th1Th17 *vs. *** **Th1**

**Gene symbol**	**Fold change Th1Th17/Th1**	**p-value**	**Gene title**
XCL1	−7,53	0,001	Chemokine (C motif) ligand 1
IL5	−3,35	0,010	Interleukin 5 (colony-stimulating factor, eosinophil)
KLRK1	−2,66	0,003	Killer cell lectin-like receptor subfamily K, member 1
LAIR2	−2,57	0,004	Leukocyte-associated immunoglobulin-like receptor 2
IL4	−2,47	0,013	Interleukin 4
TIGIT	−2,36	0,001	T cell immunoreceptor with Ig and ITIM domains
CDH1	−2,35	0,000	Cadherin 1, type 1, E-cadherin (epithelial)
IL17RB	−2,33	0,005	Interleukin 17 receptor B
CXCL10	−2,31	0,046	Chemokine (C-X-C motif) ligand 10
FCRL3	−2,30	0,048	Fc receptor-like 3
IL-17RB	−2,19	0,003	Interleukin 17 receptor B
SLAMF7	−2,08	0,027	SLAM family member 7
PTK2	−2,01	0,000	PTK2 protein tyrosine kinase 2
CXCR5	−2,00	0,013	Chemokine (C-X-C motif) receptor 5
ZNF80	−1,96	0,008	Zinc finger protein 80
CADM1	−1,89	0,037	Cell adhesion molecule 1
CD109	−1,84	0,034	CD109 molecule
SERPINB6	−1,80	0,003	Serpin peptidase inhibitor, clade B, member 6
PECAM1	−1,76	0,004	Platelet/endothelial cell adhesion molecule
NFIA	−1,64	0,009	Nuclear factor I/A
TIAM2	−1,64	0,008	T-cell lymphoma invasion and metastasis 2
BCL2L14	−1,63	0,032	BCL2-like 14 (apoptosis facilitator)
ACPL2	−1,58	0,013	Acid phosphatase-like 2
IFI27	−1,57	0,026	Interferon, alpha-inducible protein 27
CCL17	−1,54	0,019	Chemokine (C-C motif) ligand 17
ALCAM	−1,54	0,009	Activated leukocyte cell adhesion molecule
H1F0	−1,50	0,020	H1 histone family, member 0
PLA2G4A	−1,49	0,030	Phospholipase A2, group IVA
HTATIP2	−1,49	0,034	HIV-1 Tat interactive protein 2, 30 kDa
SMAD2	−1,48	0,027	SMAD family member 2
ISYNA1	−1,48	0,026	Inositol-3-phosphate synthase 1
ZNF827	−1,47	0,030	Zinc finger protein 827
TAF15	−1,47	0,040	TBP-associated factor RNA polymerase II
GATA3	−1,46	0,015	GATA binding protein 3
HLA-DOA	−1,42	0,032	Major histocompatibility complex, class II, DO alpha
ZNF642	−1,40	0,025	Zinc finger protein 642
GRK5	−1,39	0,041	G protein-coupled receptor kinase 5
ZNF167	−1,38	0,022	Zinc finger protein 167
AARS	−1,37	0,021	Alanyl-tRNA synthetase
MARS	−1,36	0,041	Methionyl-tRNA synthetase
ZNF107	−1,35	0,031	Zinc finger protein 107
ZNF443	−1,35	0,022	Zinc finger protein 443

Shown are 42 differentially expressed genes (p-value <0.05 and cut-off 1.3 fold) that are downregulated in Th1Th17 *vs*. Th1 cells.

### Gene Set Enrichment Analysis (GSEA)

To identify biological processes differentially regulated in Th1Th17 *vs.* Th1 cells upon CD3/CD28 triggering, *Gene Set Enrichment Analysis* (GSEA), a knowledge based approach for interpreting genome-wide expression data [41], was conducted from the expression levels of all the probes detected. Normalized enrichment scores (NES), nominal p-values, and false discovery rates (FDR) were analyzed to systematically test three categories of gene sets from the Molecular Signatures Database (MSigDB) of the Broad Institute (Boston, MA, USA): Canonical pathways (C2), Transcription factors (C3), and Gene Ontology (C5). Among canonical pathways differentially expressed in Th1Th17 *vs.* Th1 cells (FDR < 0.05), GSEA revealed a significant enrichment in pathways including ERK-transactivation-cytoskeletal-MAPK-JNK, Nuclear receptor/transcription (RORC, RORA, PPARγ, PPARα), Circadian clock (ARNTL), leukocyte transendothelial migration, Cytokine/cytokine receptor interactions (IL-22, CCL20, IL-23R, IL-26, IL-17, CCR6, CXCR6, IL-1R1, TNFSF25, TNFSF13B, CCR2), MAPK signaling (MAP3K4, IL-1R1), p38/MAPK, IL-23, and BMAL1/CLOCK (ARNTL) (Additional file 4: Figure S2A, left panel), while gene sets including those linked to Interferon α/β signaling (IFI27, IRF8, ISG20, MX2, IFIT3, IRF9, IRF7) and proteasome (Additional file 4: Figure S2A, right panel) were found downregulated in Th1Th17 *vs.* Th1 cells. Moreover, GSEA identified that gene sets regulated by transcription factors including RORA, Oct-1, FOXO4, SMAD4, p53, FOXO1, AP1, and NF-κB were enriched in Th1Th17 *vs.* Th1 cells (Additional file 4: Figure S2B). Furthermore, GSEA revealed that biological functions including those linked to Cell adhesion, Enzyme-linked receptor protein signaling, and Receptor signaling/protein threonine kinase activity were enriched in Th1Th17 *vs.* Th1 cells, while pathways linked to different catabolic processes were downregulated (Additional file 4: Figure S2C). Together, these GSEA results point to major functional differences between Th1Th17 and Th1 cells, with Th1Th17 cells exhibiting a distinct trafficking potential and a state of superior metabolic activation, under the control of specific transcription factors such as p53, AP-1 and NF-κB, which are known key regulators of HIV replication [21,24,42]. These features, together with a decreased proteasomal activity and interferon signaling and reduced levels of the IFN-induced antiviral factors ISG20 [43], may explain preferential HIV replication in Th1Th17 *vs.* Th1 cells.

### Gene Ontology (GO) classification by biological functions of differentially expressed genes

**Figure 3:**
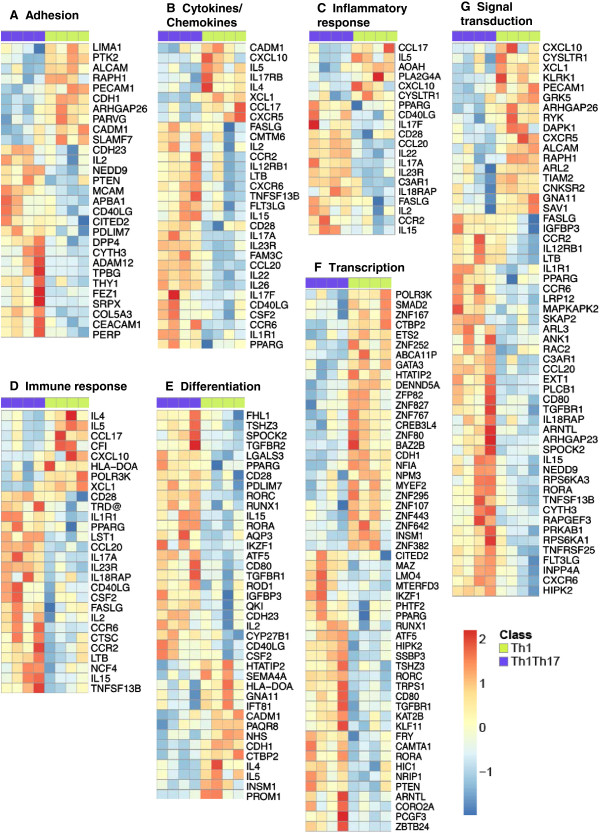
**GO classification of differentially expressed genes in Th1Th17 *vs. *Th1 cells.** Differentially expressed genes (p < 0.05, FC cut off 1.3) in Th1Th17 *vs.* Th1 were classified based on their biological functions using GO as follows: Adhesion **(A)**, Cytokines/Chemokines **(B)**, Inflammatory responses **(C)**, Immune responses **(D)**, Differentiation **(E)**, Transcription **(F)**, and Signal transduction **(G)**. The corresponding heat maps were generated using the R programming language and the pheatmap and ggplot2 libraries (R Core Team). For each heat map, genes expressed at higher and lower levels in Th1Th17 *vs.* Th1 are represented in red and blue, respectively. Results correspond to matched Th1Th17 and Th1 subsets described in Figure 2.

To extract further meaning, the differentially expressed genes in Th1Th17 *vs.* Th1 cells (p < 0.05, cut-off 1.3-fold) were classified into 13 biological functions using GO (Figure 3).

#### Adhesion molecules, cytokines, and chemokines

Genes upregulated in Th1Th17 *vs.* Th1 included the adhesion molecules MCAM (*Melanoma Cell Adhesion Molecule*) and CEACAM1 (*Carcinoembryonic Antigen cellular Adhesion Molecule 1*); the chemokine receptors CCR6, CCR2, and CXCR6; the cytokine receptors IL-1R1 and IL-12RB1 (suggested role in the differentiation and/or proliferation of IL-17 producing cells [44]); and the mRNA for cytokines such as IL-2, IL-15, IL-17A, IL-17 F, IL-22, IL-26, CCL20, lymphotoxin beta (LTB), TNFRSF13B (TNF superfamily member B cell-activating factor (BAFF) or B-lymphocyte stimulator (Blys)), and CSF2/GM-CSF (Figure 3A-B; Table 1; Additional file 2: Table S1). Genes upregulated in Th1 *vs.* Th1Th17 cells included those encoding for the adhesion molecules ALCAM (*Activated Leukocyte Cell Adhesion Molecule*), PECAM-1 (*Platelet Endothelial Cell Adhesion Molecule 1*), CDH1 (cadherin-1/E-cadherin, a ligand for KLRG1), and CADM1 (*Cell-Adhesion Molecule 1*); the surface antigen Thy-1, protein tyrosine kinase 2 (PTK2/FAK); the chemokine receptor CXCR5; the chemokines CCL17, CXCL10, and XCL1/lymphotactin; and the cytokines IL-5 and IL-4 (Figure 3A-B; Table 2; Additional file 3: Table S2). Thus, in addition to their Th1 polarization profile reflected by the expression of the Th1-specific transcription factor T-bet and the production of IFN-γ [31], the pool of CXCR3^+^CCR6^-^ T-cells includes subsets that share phenotypic and functional properties with Tfh and Th2 cells. However, levels of IL-5 production and GATA3 expression are significantly lower compared to CCR4^+^CCR6^-^ Th2 cells [31], suggesting that the frequency of Th2 cells within the CXCR3^+^CCR6^-^ fraction is relatively low. Together these results reveal distinct molecular mechanisms involved in the regulation of tissue-specific trafficking for Th1Th17 (*via* MCAM, CEACAM1, CCR2, CXCR6) and Th1 cells (*via* ALCAM, PECAM-1, CDH1, CADM1, and CXCR5) that may be linked to the distinct *in situ* localization and contribution of these subsets to HIV pathogenesis.

#### Immune responses and inflammation

Th1Th17 cells express higher levels of transcripts corresponding to the costimulatory molecules CD28, CD40 ligand (CD40LG/CD154), FAS ligand (FASLG), the cytokines IL-2 and IL-15, LST1 (leukocyte specific transcript 1, a regulator of cell motility [45]), the cytokines receptor IL23R (whose ligand IL-23 is involved in the stabilization of Th17 polarization [46] and the induction of pathogenic Th17 cells [47]), and the IL-18 receptor accessory protein (IL18RAP) (Figure 3C-D, Additional file 2: Table S1). In contrast, Th1 *vs.* Th1Th17 cells expressed superior RNA levels corresponding to the chemokines CCL17, CXCL10, XCL1 and the cytokines IL-5 and IL-4 (Figure 3C-D, Additional file 3: Table S2). Thus, Th1Th17 *vs.* Th1 cells appear to be more susceptible to activation, cell proliferation, and apoptosis, and also prone to respond to IL-23 for the maintenance of their Th17 differentiation and pathogenic potential.

#### Transcription and cell differentiation

Genes expressed at higher levels in cells corresponding to the Th1Th17 profile include the costimulatory molecule CD80, the receptors for TGF-β, TGFBR1 and TGFBR2, the Th17-specific transcription factors RORC and RORA [48,49], RUNX1 (runt-related transcription factor 1), the nuclear receptor *Peroxisome Proliferator-Activated Receptor Gamma* (PPARγ), ATF5 (*Activating Transcription Factor 5*), KLF11 (*Kruppel-like factor 11*), PTEN (*phosphate and tensin homolog)*, and ARTNL (*Aryl Hydrocarbon Receptor Nuclear Translocator-Like, a* tumor suppressor and negative regulator of the cell cycle [50])) (Figure 3E-F; Table 1; Additional file 2: Table S1). In contrast, Th1 cells preferentially express the mRNAs corresponding to the signal transducer SMAD2 (mediating signals related to TGF-β [51]), the transcription factor GATA3 (associated to the Th2 polarization profile [52]), as well as 10 members of the zinc finger (ZNF) family, some of which bind DNA and repress transcription of multiple genes [53,54]: ZNF80, ZNF82, ZNF107, ZNF167, ZNF252, ZNF295, ZNF382, ZNF443, ZNF642, ZNF767 and ZNF827 (Figure 3F; Table 2; Additional file 3: Table S2). Consistent with previous findings by our group and others [30,31], the current microarray studies demonstrate that Th1 and Th1Th17 cells express similarly high levels of the Th1-specific transcription factor T-bet (data not shown). Thus, Th1Th17 and Th1 subsets express distinct transcription factors that control their differentiation, polarization and biological functions by regulating the transcription of distinct genes that may play a role in HIV permissiveness.

#### Signal transduction

The heat map of genes implicated in signal transduction shows the differential expression of different kinases that can influence the sensibility to various signals. TCR triggering in Th1Th17 *vs.* Th1 cells induced higher expression of mRNA corresponding to kinase MAPKAPK2/MK2 (mitogen-activated protein kinase-activated protein kinase 2, important for the sustained NF-κB activation [55]), RAPGEF3/Epac1 (an exchange protein activated by cAMP, its expression could promote cell adhesion [56]) and the homeodomain interacting protein kinase 2 (HIPK2, positive regulator of the tumor suppressor p53 [57]). In contrast, Th1 cells preferentially express the mRNA of the kinases GRK5 (that inhibits the NF-κB transcriptional activity by inducing nuclear accumulation of IκBα [58,59]), and CNKSR2/KSR2 (kinase suppressor of Ras 2, another negative regulator the NF-κB pathway [60]) (Figure 3I; Tables 1 and 2; Additional file 2: Table S1 and Additional file 3: Table S9). Of note, Th1Th17 express the killer cell lectin-like receptor subfamily B, member 1 (KLRB1/CD161) (Table 1), a known marker for Th17 precursors [61], while Th1 cells express the killer cell lectin-like receptor subfamily K, member 1 (KLRK1) (Figure 3G; Table 2). Thus, compared to Th1Th17 cells, Th1 polarized cells appear less favorable to activation and signal transduction of the NF-κB pathway, a signaling pathway implicated in HIV replication [21,22].

##### RT-PCR validation of microarray results

**Figure 4:**
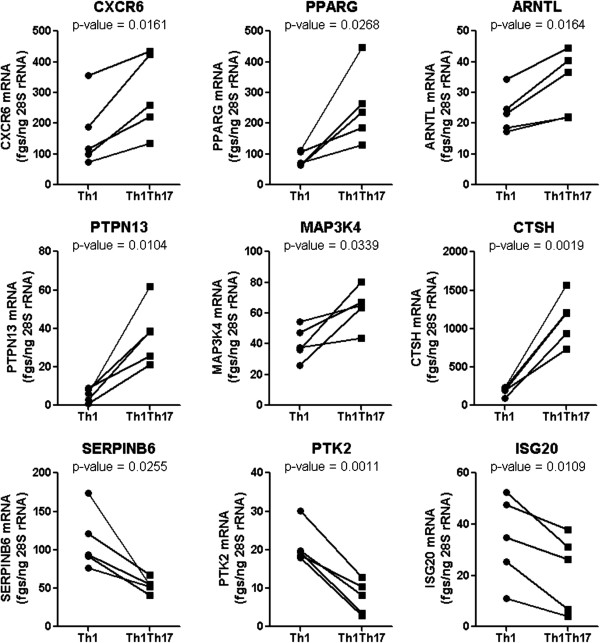
**RT-PCR validation of differentially expressed genes.** Total RNA was extracted from CD3/CD28-stimulated matched Th1Th17 and Th1 subsets. The expression of CXCR6, PPARγ, ARNTL, PTPN13, MAP3K4, CTSH, SERPINB6, PTK2, and ISG20 mRNA was quantified by SYBR green real time RT-PCR. Quantification was performed relative to a standard curve generated based on cDNA specific for each transcript. The expression of each gene was normalized to the 28S rRNA internal control and expressed as fgs RNA of a target gene per 1 ng 28S rRNA. Depicted are results obtained with matched Th1Th17 *vs.* Th1 cells from five different HIV-uninfected individuals. Paired *t*-Test values are indicated on the graphs.

Among genes differentially expressed in matched Th1Th17 *vs.* Th1 cells (p-value <0.05 and FC cutoff 1.3) (Tables 1 and 2), a number of nine genes were selected by intelligent guess for validation at mRNA level. Among these transcripts, CXCR6 [62,63], PPARγ [64-66], SERPINB6 [22], ARNTL [24], PTK2/FAK [67,68]), and ISG20 [43] were previously reported to play a role in regulating HIV replication, while PTPN13/FAP-1 [69-71] and MAP3K4/MEKK4 [72-74] are linked to T-cell activation. Quantitative real-time RT-PCR demonstrate that transcripts for CXCR6, PPARγ, ARNTL, PTPN13, MAP3K4, and CTSH were significantly upregulated in Th1Th17 *vs.* Th1, while SERPINB6, PTK2, and ISG20 were downregulated (Figure 4). These results validated the differential expression of a set of transcripts in Th1Th17 *vs.* Th1 cells at mRNA level and suggest a potential role played by these molecules in the regulation of HIV permissiveness/resistance.

##### CEACAM1 and MCAM are new surface markers for Th1Th17 cells

We further sought to validate the expression of two adhesion molecules at the protein level using flow cytometry: CEACAM1, a tumor marker [75] and broad inhibitor of T-cell function [76] and MCAM, a Th17 marker [77] and regulator of cell recruitment into the brain [78]. Results in Additional file 5: Figure S3 demonstrate a significantly higher expression of CEACAM1 and MCAM on Th1Th17 *vs.* Th1 cells *ex vivo*. Thus, CEACAM1 and MCAM are two new surface markers for Th1Th17 cells likely involved in regulating the *in situ* localization and function of these cells, with potential relevance for HIV pathogenesis.

##### The transcription repressor PPARγ is preferentially expressed in Th1Th17 cells

**Figure 5:**
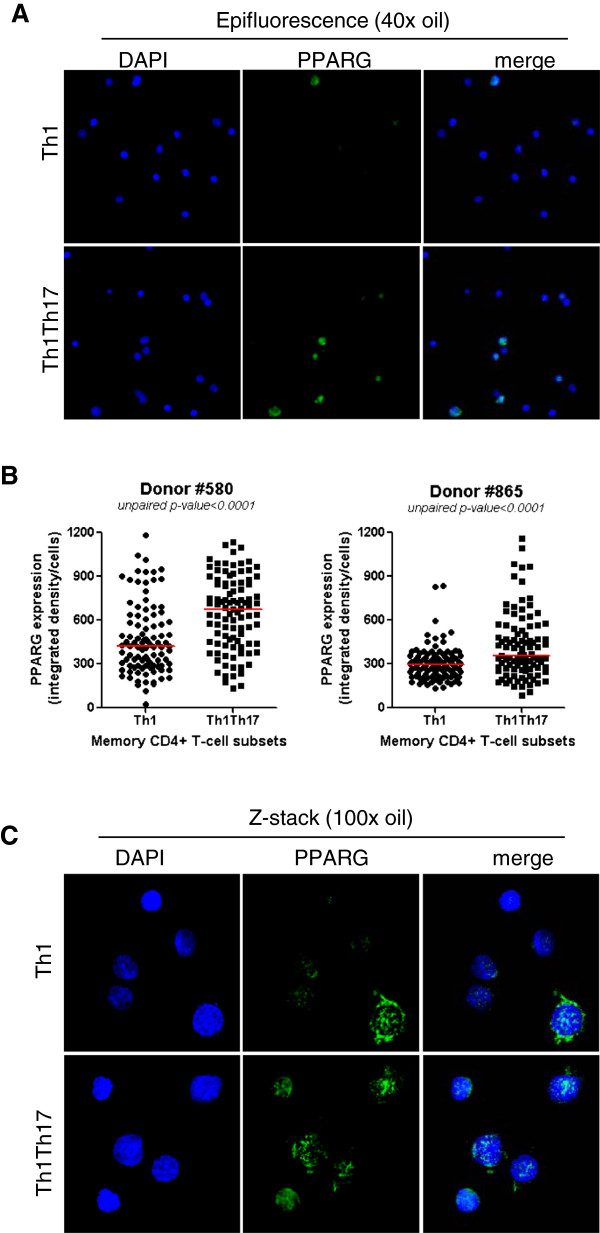
**Confocal microscopy quantification of PPARγ expression in Th1Th17 *vs. *Th1 cells.** Matched Th1Th17 and Th1 cells were stimulated *via* CD3/CD28 for 3 days. Cells were fixed on poly-L-lysine coated slides and intracellular staining was performed with rabbit anti-human PPARγ Abs and then AlexaFluor 488 goat anti-rabbit Abs. Slides were mounted with the *ProLong Gold Antifade* reagent containing the nuclear dye DAPI. Slides were observed by fluorescence microscopy. **(A-B)** PPARγ expression was observed by epifluorescence at 40x magnification. **(A)** Shown are fields of Th1 and Th1Th17 cells from one donor representative of two donors. **(B)** Shown is statistical analysis of PPARγ expression in Th1 *vs.* Th1Th17 cells in two different donors (n = 100 cells *per* subsets *per* donor). Horizontal red lines indicate median values. Unpaired p-values are indicated on the figures. **(C)** The intracellular localization of PPARγ was observed by maximum intensity z-projection of z-stack from representative field of each subset observed with a 100x oil objective in a spinning-disc confocal mode system. Shown images are representative of observations made with Th1 and Th1Th17 from two different donors.

The nuclear receptor PPARγ is a transcription factor known to inhibit Th17 differentiation in mice [79] and humans [80]. Here, we used fluorescence and confocal microscopy to validate differential expression of PPARγ protein in human Th1Th17 *vs.* Th1 cells at day 3 post CD3/CD28 triggering. Despite a marked heterogeneity in PPARγ expression within each subset, the expression of PPARγ protein was significantly higher in Th1Th17 *vs.* Th1 cells in two independent donors (Figure 5A-B). The analysis of cellular localization of PPARγ by confocal microscopy and z-stack reconstruction demonstrated the expression of PPARγ in both nuclear and cytoplasmic compartments and a tendency for superior nuclear expression of PPARγ in Th1Th17 *vs.* Th1 cells (Figure 5C). These results demonstrate an increased frequency of cells expressing PPARγ within human Th1Th17 *vs.* Th1 subsets and provide evidence that a partial PPARγ nuclear translocation occurs in Th1Th17 upon TCR triggering.

##### PPARγ negatively regulates HIV replication in Th1Th17 cells

**Figure 6:**
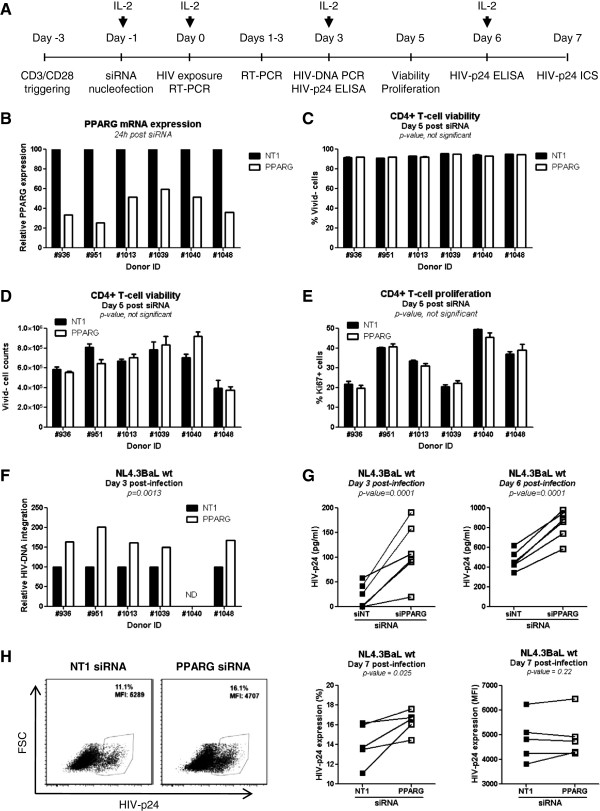
**PPARγ RNA interference increases HIV replication in memory CD4**^**+ **^**T cells.** Memory CD4^+^ T-cells were isolated from thawed PBMC rested overnight by negative selection using magnetic beads. **(A)** Cells were stimulated *via* CD3/CD28 for 2 days, nucleofected with PPARγ or non-targeting (NT1) siRNA, and then cultured for an additional 24 h at 2x10^6^ cells/ml in the presence of IL-2. **(B)** RNA silencing efficiency was assessed by RT-PCR 24 h post-nucleofection (n = 6). **(C-E)** The effect of NT1 and PPARγ siRNA on cell viability and proliferation was assessed by flow cytometry at day 6 post-nucleofection (n = 6). Cell viability was monitored using the viability dye Vivid **(C)**, cell counts were determined using FlowCounts fluorospheres **(D)**, and cell proliferation was measured upon intracellular staining with Ki67 Abs **(E)**. Cells were exposed to replication competent NL4.3BAL wt strain (10 ng HIV-p24/10^6^ cells) for 3 h at 37°C. Unbound virus was removed by extensive washing, and cells were maintained in culture in the presence of IL-2 up to 7 days post-infection. **(F)** Integrated HIV-DNA levels were quantified by real-time PCR in cells harvested at day 3 post-infection (n = 5). **(G)** Levels of HIV-p24 in cell supernatants were quantified by ELISA at days 3 and 6 post-infection (n = 6). **(H)** At day 7 post-infection, cells were harvested and the frequency of infected cells was analyzed by flow cytometry upon intracellular staining with HIV-p24 Abs. Shown is the frequency of HIV-p24^+^ cells (% and MFI, mean fluorescence intensity) upon nucleofection with PPARγ *vs.* NT1 siRNA in one representative subject **(left panels)** and statistical analysis in n = 5 different subjects **(right panels)**. Paired *t*-Test values are indicated on the graphs.

The PPARγ activation pathway restricts HIV replication in macrophages [64,66]. To investigate the role of PPARγ in regulating HIV permissiveness in Th1Th17 cells, we first used RNA interference to reduce levels of PPARγ mRNA prior HIV exposure. Given the difficulty of sorting sufficient numbers of Th1Th17 cells, siRNA experiments were performed on TCR-activated total memory CD4^+^ T-cells from n = 6 different donors (Figure 6A). The efficacy of PPARγ mRNA and protein silencing was measured by RT-PCR and microscopy, respectively, at 24 h, 48 h, and 72 h post-nucleofection. In parallel, cells were exposed to the wild type (wt) NL4.3BaL R5 HIV strain or the single round HIV-VSVG-GFP strain at 24 h or 72 h post-nucleofection. Viral replication was monitored up to day 6 post-infection. Levels of PPARγ mRNA were significantly decreased in 6/6 donors at 24 h post-nucleofection (range silencing: 40-75%) (Figure 6B). However, epifluorescence analysis (40X magnification) revealed a significant decrease in the PPARγ protein expression at 48 h (2/3 subjects) and 72 h (3/3 subjects) but not at 24 h post-nucleofection (Additional file 6: Figure S4A). The analysis of cellular localization of PPARγ by confocal microscopy and z-stack reconstruction demonstrated a significant depletion of the pool of PPARγ protein from both cytoplasm and nuclei at 72 h post-nucleofection (Additional file 6: Figure S4B). Cell viability (% and counts of Vivid^-^ cells) and proliferation (% Ki67^+^ cells) was not significantly different when nucleofection was performed using NT1 *vs.* PPARγ siRNA (Figure 6C-E), suggesting that subsequent differences in HIV permissiveness between NT1 and PPARγ siRNA are not linked to differences in cell toxicity caused by nucleofection. When cells were exposed to the replication competent NL4.3BaL 24 h post-nucleofection, PPARγ silencing was associated with a significant increase in HIV-DNA integration in 5/5 subjects at day 3 post-infection (Figure 6F; range increase: 150-200%). In addition, PPARγ knock down resulted in enhanced viral replication as reflected by the HIV-p24 levels in supernatants at days 3 and 6 post-infection (Figure 6G) and the frequency of HIV-p24^+^ cells at day 7 post-infection (Figure 6H). Thus, PPARγ restricts HIV replication in CD4^+^ T-cells.

**Figure 7:**
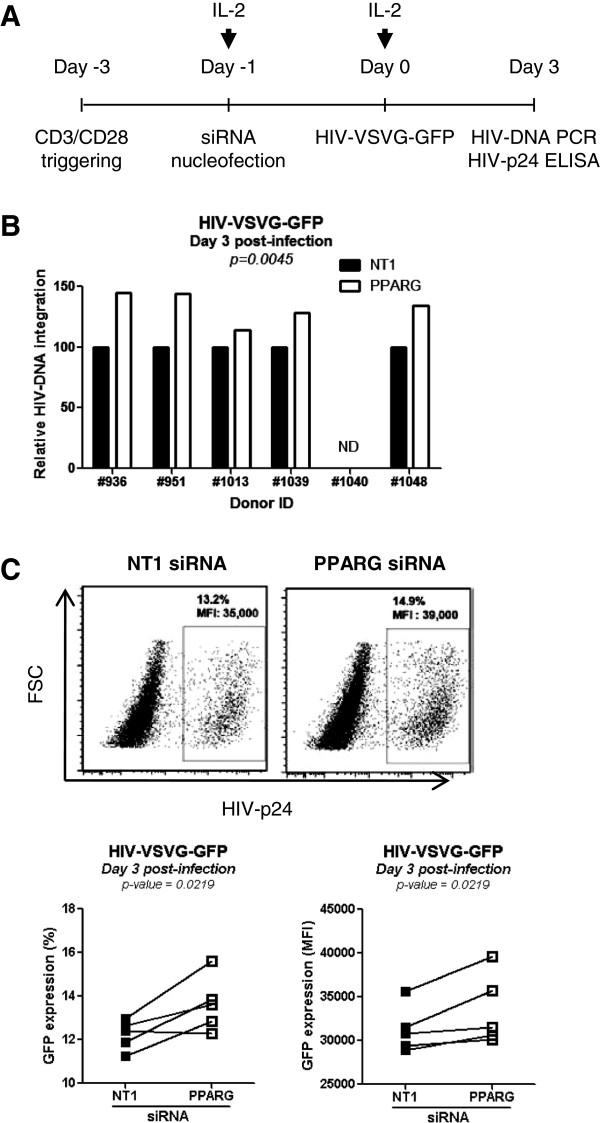
**PPARγ pathway negatively regulates HIV replication in T-cells at levels post-entry and prior integration. (A)** CD4^+^ T-cells were isolated, activated *via* TCR and nucleofected with PPARγ *vs.* NT1 siRNA as described in Figure 6. Cells were exposed to single round HIV-VSVG-GFP at 24 h post-nucleofection and cultured for three days in the presence of IL-2. **(B)** Shown are levels of integrated HIV-DNA (n = 5). **(C)** Shown is the frequency and MFI of GFP-expressing cells in one representative subject **(upper panels)** and statistical analysis in n = 5 different subjects **(lower panels)**. Paired *t*-Test values are indicated on the graphs.

To determine at which step in the viral life cycle PPARγ interferes with HIV replication, similar experiments were performed with cells exposed to the single round HIV-VSVG-GFP strain 24 h post-nucleofection with NT1 or PPARγ siRNA (Figure 7A). Results demonstrated that during one round of replication PPARγ knock down leads to a significant increase in HIV-DNA integration (Figure 7B) and transcription, as reflected by superior % and MFI GFP expression (Figure 7C). Despite a marked PPARγ protein knock down at 72 h upon nucleofection, cell exposure to HIV at this time point reveal only minor differences in HIV replication between NT1 and PPARγ siRNA (data not shown). This prompted us to investigate the kinetics of PPARγ mRNA expression upon nucleofection. Results in Additional file 7: Figure S5 reveal the recovery of PPARγ mRNA pool 72 h post-nucleofection. This may lead to the subsequent recovery of the PPARγ protein pool, thus explaining why differences in viral replication are not observed anymore when cell exposure to HIV is performed 72 h post-nucleofection. Altogether, these results identify PPARγ as a negative regulator of HIV integration and replication in CD4^+^ T-cells by acting at levels post-entry and prior HIV-DNA integration.

**Figure 8:**
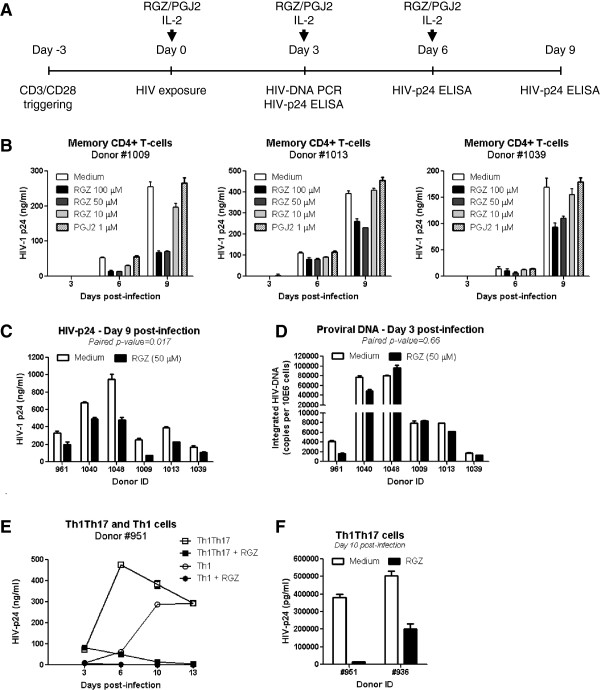
**Activation of PPARγ pathway negatively regulates HIV replication in T-cells.** Memory CD4+ T cells were stimulated *via* CD3/CD28 for 3 days. **(A)** Cells were then exposed to replication competent NL4.3BAL-GFP HIV (50 ng HIV-p24/106 cells) for 3 h at 37°C. Unbound virus was removed by extensive washing. HIV-infected cells were cultured in the presence of IL-2 (5 ng/ml) and Rosiglitazone (RGZ; 10, 50, and 100 μM) or prostaglandin J2 (PGJ2; 1 μM). Media was refreshed every 3 days. Supernatants were harvested at day 3, 6 and 10 post-infection for HIV-p24 quantification by ELISA, while a fraction of cells harvested at day 3 post-infection was used for integrated HIV-DNA quantification by real-time PCR. **(B)** Shown are kinetics of HIV-p24 expression in culture supernatants (n = 3; mean±SD of triplicate wells). **(C)** Shown are statistics of HIV-p24 levels in supernatants collected at day 9 post-infection from memory CD4+ T-cells cultured in the presence or the absence of RGZ (50 μM) (n = 6). **(D)** Shown are integrated HIV-DNA levels at day 3 post-infection in memory CD4+ T-cells cultured in the presence or the absence of RGZ (50 μM) upon infection (n = 6). **(E)** Matched Th1Th17 and Th1 cells were sorted by flow cytometry, stimulated and exposed to NL4.3BAL-GFP HIV as above, and cultured with IL-2 (5 ng/ml) in the presence or absence of RGZ (50 μM). Levels of HIV-p24 were quantified by ELISA at days 3, 6, 10, and 13 post-infection. Shown are results from one donor representative of results obtained with cells from two different donors. **(F)** Shown are the effects of RGZ (50 μM) on HIV replication in Th1Th17 cells from two different donors at day 10 post-infection. **(C-D)** Paired *t*-Test values are indicated on the graphs (n = 6).

To further investigate the role of the PPARγ activation pathway in controlling HIV replication in T-cells, we used two PPARγ agonists: the synthetic rosiglitazone (RGZ) and the natural prostaglandin J2 (PGJ2). The PPARγ-specific antagonist T007907 [81] was also used to counteract the effects of RGZ. Several doses of RGZ and PGJ2 were tested for their effects on cell viability and proliferation and on the expression of HIV receptor CD4 and coreceptor CCR5. RGZ at 1–100 μM and PGJ2 at 1–10 μM had no deleterious effects on cell viability and proliferation, and did not alter CD4 expression (Additional file 8: Figure S6A-D). RGZ did not alter CCR5 expression, while PGJ2 at 5 and 10 but not 1 μM increased CCR5 expression (Additional file 8: Figure S6E), suggesting an unexpected facilitation of HIV entry *via* CCR5 by PGJ2. Doses of RGZ and PGJ2 that did not interfere with cell viability, proliferation, and CD4/CCR5 expression were used in subsequent experiments. Confocal microscopy visualization of cells treated with RGZ (50 μM), demonstrated a massive translocation of PPARγ from the cytoplasm to the nucleus; this process was highly specific as it was efficiently reversed by simultaneous exposure to the antagonist T007907 (Additional file 9: Figure S7A-B). To determine the effect of PPARγ pathway activation on HIV replication, total memory CD4^+^ T-cells were first exposed to HIV and then cultured in the presence or absence of RGZ (10, 50, 100 μM) and PGJ2 (1 μM) (Figure 8A). Treatment with RGZ at 50 and 100 but not 10 μM significantly reduced HIV replication at day 6 and 9 post-infection, while the PGJ2 at 1 μM had no significant effect (Figure 8B-C). The quantification of HIV-DNA integration at early time points (day 3 post-infection) in cells treated with RGZ after HIV exposure did not reveal statistically significant differences (Figure 8D), suggesting that PPARγ pathway activation limits HIV replication by interfering with viral transcription. However, RGZ significantly decreased HIV-DNA integration at late time points (day 14 post-infection) (data not shown), suggesting that PPARγ pathway activation limits HIV dissemination upon long-term treatment. Finally, the role of PPARγ pathway in the negative regulation of HIV replication was tested in sorted Th1Th17 and Th1 cells from two independent donors. Results in Figure 8E-F illustrate that RGZ treatment dramatically reduced HIV replication in Th1Th17. RGZ also limited HIV replication in Th1 cells Figure 8D, consistent with the fact that cells expressing PPARγ are also detected within the pool of Th1 cells (Figure 5). Together these results provide the first evidence that PPARγ acts as an intrinsic negative regulator of HIV replication in CD4^+^ T-cells, including cells with a Th1Th17 polarization profile. PPARγ prevents new infection by acting at levels prior HIV-DNA integration. PPARγ also acts on infected cells and limits replication and subsequent infection spreading to neighboring cells by acting at post-integration levels, likely during transcription.

## Discussion

The goal of this study was to identify molecular mechanisms of HIV-1 regulation in Th1Th17 and Th1 cells, two cell subsets previously identified by our group as being highly permissive and relatively resistant to infection, respectively [31]. We performed a genome-wide analysis of gene expression in Th1Th17 and Th1 cells upon TCR signaling and prior HIV exposure. Our results demonstrate that *(i)* Th1Th17 cells have the potential to be recruited into sites of HIV persistence such as the intestine and the brain; *(ii)* pathways previously linked to HIV permissiveness, including the proximal TCR signaling and the NF-κB activation pathway [21-26] are enriched in Th1Th17 *vs.* Th1 cells; and *(iii)* the transcriptional repressor PPARγ is an intrinsic negative regulator of HIV permissiveness in total CD4^+^ T-cells and mainly Th1Th17 cells by acting at post-entry levels, prior to HIV-DNA integration and/or transcription. Together, these results reveal that Th1Th17 cells exhibit a unique transcriptional program favorable to HIV replication that can be negatively regulated by the nuclear receptor PPARγ.

Genome-wide transcriptional profiling demonstrated that, despite a high degree of transcriptional similarity, Th1Th17 and Th1 cells are distinguished by the differential expression of 780 probe sets (p-value <0.05), including 265 upregulated and 235 downregulated, respectively (FC cutoff 1.3). Transcripts differentially expressed in Th1Th17 *vs.* Th1 cells were classified using two systematic approaches: GSEA and GO. GSEA demonstrated that transcripts linked to p38 MAPK signaling, integrins, transendothelial migration, cytokine receptor interaction, IL-23 signaling, and circadian clock pathways were enriched in Th1Th17 cells. In contrast, transcripts linked to interferon type 1 signaling pathway, proteasome, and cell cycle were enriched in Th1 cells. Also, transcripts with promoters controlled by transcription factors such as RORA, Foxo1, Foxo4, AP-2, NF-κB, and p53 were enriched in Th1Th17 *vs.* Th1 cells. GO classification by biological function revealed significant differences between Th1Th17 and Th1 cells relative to transcripts involved in cell adhesion, cytokines/chemokines, inflammatory processes, immune responses, differentiation, transcription, and signal transduction. The finding that the NF-κB activation pathway, known for its implication in enhancing the transcription of different genes including the HIV LTRs [82], was enriched in Th1Th17 is consistent with results generated by siRNA screens for HIV-dependency factors in cell lines [21,24]. Several Th1Th17-specific transcripts were also recently identified by Imbeault et al. as being over expressed in HIV-infected *vs.* uninfected CD4^+^ T-cells [42]. These transcripts are linked to T-cell polarization (cytokines and transcription factors specific for Th17 and Th1 cells) and activation (CD80, CD40LG, NF-κB, Rho/Rac, RUNX1), susceptibility to apoptosis (FasLG, PTEN, p53), and regulation of viral replication (furin) [42]. Finally, our finding that gene sets linked to interferon type I signaling, including the antiviral factor ISG20 [43], are over expressed in Th1 cells is consistent with the up regulation of these pathways in HIV-resistant CMV-specific CD4^+^ T-cells [36]. Thus, we provide evidence that HIV permissiveness in Th1Th17 *vs.* Th1 is associated with a superior state of cellular activation and limited antiviral properties.

Our study reveals that trafficking of Th1Th17 and Th1 cells is regulated by distinct adhesion molecules and chemokine receptors: CCR6, MCAM, CEACAM1, CCR2, and CXCR6 for Th1Th17 and PEACAM-1/CD31, ALCAM, and CXCR5 for Th1. CCR6 is a Th17 marker essential for their homing into Peyer’s patches [83] and the central nervous system (CNS) [84]). MCAM facilitates entry into the CNS of T-cells involved in the pathogenesis of multiple sclerosis [78,85]. CCR2 is another Th17 marker [86] that also acts as minor co-receptor for HIV entry [87]). A polymorphism in CCL2 (a major CCR2 ligand) leading to increased chemokine expression was associated with AIDS dementia [88]. The CNS is a major HIV sanctuary established during the early phases of infection [3,89]. Recruitment of Th1Th17 cells into the brain *via* CCR6, MCAM, and CCR2 may significantly contribute to fueling HIV persistence. CEACAM1 expression is induced by activation [76] and plays a critical role in the regulation of B cell function at intestinal level [90]. The functional role of CEACAM1^+^ Th1Th17 cells in gut-associated lymphoid tissues (GALT), a major anatomic site for HIV replication [91], remains to be determined. CXCR6 is co-expressed with CCR5 on activated T-cells [63] and acts as a minor HIV coreceptor [92]. CXCR6 is also involved in the formation of the immunological synapse upon interaction with its ligand CXCL16, a membrane-bound chemokine expressed by DC [62,93]. This interaction my lead to efficient transmission of HIV within the virological synapse [94]. CXCL16 is also expressed by CD16^+^ monocytes [95]. The pro-inflammatory CD16^+^ monocytes are expanded in HIV-infected subjects [96], carry integrated HIV-DNA *in vivo*[97] and exhibit the ability to promote viral replication in CD4^+^ T cells *in vitro*[37,38]. Thus, the interaction of Th1Th17 cells with DC or CD16^+^ monocytes *via* CXCR6-CXCL16 may contribute significantly to cell-to-cell transmission of HIV *in vivo*. Consistent with this prediction, the CXCR6 polymorphism was associated with slow disease progression in HIV-infected people [92,98]. The fact that Th1Th17 cells express receptors regulating both HIV entry and migration into anatomic sites of viral replication place these cells in the first line of HIV targets and provides an explanation for their depletion in HIV-infected subjects, including subjects under viral-suppressive ART [31].

The autocrine production of CCR5-binding chemokines protects CD4^+^ T-cells from HIV infection [32,33]. Our microarray studies did not reveal differences between Th1Th17 and Th1 cells in the expression of these transcripts, consistent with our previous results [31]. However, the upregulation in Th1 *vs.* Th1Th17 cells of the PTK2/FAK, a kinase whose activation is linked to CCR5 triggering [67,68], suggests that CCR5-binding chemokines act on Th1 cells and may limit this way HIV entry. This scenario is consistent with the finding that differences in HIV-DNA integration were marginally significant when cells where exposed to a single round VSV-G-pseudotyped HIV. Nevertheless, levels of GFP expression, indicative of HIV transcription, were higher in Th1Th17 *vs.* Th1 cells suggesting that regulatory mechanisms at both entry and post-entry levels control HIV permissiveness in Th1Th17 *vs.* Th1 cells.

Of particular interest, Th1 cells expressed transcripts corresponding to KLRK1/NKG2D, an activating receptor typically expressed on cytotoxic NK cells and CD8^+^ T-cells [99]. The acquisition of cytotoxic antiviral function was previously reported for CMV-specific cells [100], which exhibit indeed a Th1 polarization profile [30] and are protected from infection [32,33,36]. The acquisition of cytotoxic antiviral functions may contribute to the intrinsic HIV resistance in Th1 cells thus, explaining the expansion of Th1 cells in HIV-infected subjects [31]. An increased frequency of Th1 cells in HIV-infected subjects may be deleterious given the ability of these cells to produce pro-inflammatory cytokines such as TNF [31]. In addition, the finding that Th1 *vs.* Th1Th17 cells over expressed the CDH1 mRNA (E-cadherin), known to inhibit HIV-specific CD8^+^ T-cell functions *via* interaction with its receptor KLRG1 [101], suggests a deleterious role of Th1 cells in HIV pathogenesis.

In addition to known Th17-specific transcription factors (RORC, RORA [48,49]), we demonstrate that Th1Th17 *vs.* Th1 expressed at superior levels the transcription factors RUNX1, known to mediate the transactivation of RORC [102]; ATF5, involved in T cell activation [103]; ARTNL, a component of the circadian clock [104] and an HIV dependency factor [24]; PPARγ [105-107]; and also KLF11, which is a PPARγ co-regulator and direct transcriptional target [108]. HIV permissiveness in Th1Th17 cells was also associated with superior expression of the costimulatory molecules CD28 and CD40LG/CD154, molecules involved in the regulation of apoptosis such as FASLG, and cytokines such as IL-2 and IL-15. The expression of TGFBR1/TGFBR2 and IL23R on Th1Th17 cells is indicative of their ability to respond to TGF-β and IL-23, respectively. TGF-β is essential to Th17 differentiation [47,109], while IL-23 is involved in the maintenance of the Th17 polarization [46] and critical for the development of a pathogenic Th17 profile [47].

The expression of genes important for signal transduction downstream of the TCR and/or cytokine receptors also indicates a greater susceptibility of Th1Th17 cells to activation and a potential contribution to inflammation. For example, Th1Th17 cells preferentially express MAPKAPK2/MK2, a kinase involved in the production of TNF-α and IL-6 [110] and MAP3K4, a kinase involved the p38/JNK pathway activation in response to TGF-β [74]. Indeed, signaling through p38 is important for HIV replication [111]. In contrast to Th1Th17, Th1 cells expressed at superior levels several 10 genes of the zinc finger (ZNF) family, including the ZNF382, a tumor suppressor acting *via* the inhibition of the NF-κB signaling pathway [112]. Also, Th1 cells preferentially express GRK5 and CNKSR2/KSR2, two molecules that can inhibit the transcriptional activity of NF-κB [58,60]. Interestingly, genome-wide siRNA screens for HIV dependency factor (HDF) identified NF-κB pathway as being key for HIV permissiveness [21,22,24]. It was also shown that the HIV LTR promoters have binding sites for NF-κB that are important for the transcription of the virus [82]. It would be of interest to determine whether the expression of kinases GRK5 and CNKSR2 limits NF-κB translocation in Th1 cells thus, explaining their resistance to HIV infection.

One major finding of our study is the identification of PPARγ as an intrinsic negative regulator of HIV permissiveness in Th1Th17 cells. PPARγ is a ligand-dependent nuclear receptor that acts as a transcriptional repressor in macrophages [105] and T-cells [106,107]. The localization of PPARγ in the nucleus *vs.* the cytoplasm is influenced by the interactions with its endogenous ligands [107]. Confocal microscopy analysis revealed not only an increased frequency of PPARγ expressing cells, but also superior nuclear *vs.* cytoplasmic localization of PPARγ protein in Th1Th17 *vs.* Th1 cells, suggesting the existence of endogenous ligands triggering PPARγ nuclear translocation in Th1Th17 cells upon TCR engagement. Whether PPARγ^+^*vs.* PPARγ^−^ cells within the Th1Th17 and also the Th1 pool are particularly permissive to infection and whether the nuclear localization of PPARγ contribute to limiting HIV permissiveness in such cells remains unknown. Future studies are needed to determine the role of PPARγ endogenous ligands in controlling HIV permissiveness in primary cells.

Our results demonstrate that PPARγ activation pathway controls HIV dissemination by acting on HIV-infected cells and also by preventing new integrative infection. We found that siRNA against PPARγ led to a significant increase in HIV-DNA integration and subsequent viral replication when cells were exposed to wt HIV 24 h after PPARγ knock down. Of note, similar results were obtained when cells were exposed to single round VSV-G pseudotyped virions that enter cells independently of CD4 and coreceptors. Thus, we provide evidence that PPARγ exerts its inhibitory effects post-entry and prior HIV-DNA integration. The activation of the PPARγ pathway using the synthetic agonist RGZ upon HIV exposure demonstrated a strong inhibition of HIV replication. Consistent with previous studies on dendritic cells (DC) [113], RGZ did not affect the expression of CD4 and CCR5 thus providing further evidence that PPARγ activation interferes with HIV replication at post-entry levels. Treatment with RGZ induced a complete nuclear translocation of PPARγ, and this phenomenon was reversed by simultaneous exposure to the antagonist T007907. The effects of RGZ on HIV-DNA integration was observed at late but not early time points post-treatment thus, suggesting that nuclear translocation of PPARγ in HIV-infected cells limits viral replication by regulating directly or indirectly HIV transcription and subsequent HIV dissemination. Unexpectedly, the natural PPARγ agonist PGJ2 exerted no effect on HIV replication, in contrast to previous studies on different cell types [66,114], suggesting that PGJ2 effects are cell-dependent. One potential explanation is related to the fact that PGJ2 exerts PPARγ-independent effects [115]. Another explanation may be that Th1Th17 selectively express transcripts for hydroxyprostaglandin dehydrogenase 15-(NAD) (HPGD) (Table 1), an enzyme involved in PGs degradation [116]. Whether, HPGD degrades exogenous PGJ2 remains unknown. Finally, we observed that RGZ dramatically decreased HIV replication in sorted Th1Th17 cells thus demonstrating that PPARγ indeed negatively controls the HIV permissiveness program in these cells. The ability of RGZ to control HIV replication in Th1Th17 cells but also in total CD4^+^ T-cells is of interest in view of future therapies targeting PPARγ nuclear translocation *in vivo*.

Other studies demonstrated the ability of PPARγ agonists to inhibit HIV replication *in vitro*[66,113,114] or in animal models of HIV encephalitis by acting on macrophages [64]. Also, PPARγ signaling inhibits DC-mediated HIV capture and *trans*-infection at least in part by depleting cholesterol from the cell membrane [113]. Of note, two negative regulators of the PPARγ signaling, NCOR and COUP, were previously identified as HDFs in siRNA screenings performed in HeLa [21] and Jurkat cells [24], respectively. Our results provide the first evidence that HIV-permissive Th1Th17 cells highly express PPARγ, which acts as an intrinsic negative regulator of viral replication. These effects may be explained by different indirect mechanisms including the anti-inflammatory properties of PPARγ [107,117], the alteration of the cholesterol metabolism [113,118], and/or by the ability of PPARγ to repress RORC expression and subsequently inhibit Th17 differentiation [79,80]. In addition to these indirect effects, there is evidence supporting a directed capacity of PPARγ to repress HIV LTR activity [64,119]. A very recent sequence analysis of the HIV-1 5′-LTR region in viral isolates from different geographic regions, suggests the possible conservation of PPAR binding sites [120]. Thus, PPARγ represent a robust negative regulator of HIV replication in permissive CD4^+^ T-cells, such as Th1Th17 cells, *via* both direct and indirect mechanisms of HIV integration and transcription regulation.

## Conclusions

To our knowledge, this is the first genome-wide characterization of differential gene expression in primary Th1Th17 *vs.* Th1 cells that we previously identified as being relatively permissive and resistant to HIV infection, respectively [31]. This study identifies new markers regulating Th1Th17 trafficking and functions (MCAM, CEACAM1, CXCR6), the NF-κB as a major pathway involved in the positive control of HIV permissiveness, and the PPARγ pathway as a negative regulator of HIV replication in primary CD4^+^ T-cells. PPARγ agonists, initially discovered as anti-diabetic drugs [121], are of therapeutic interest in humans given their anti-inflammatory properties. In HIV-infected subjects, PPARγ agonists are already used to treat chronic metabolic abnormalities [122]. Given our current findings on the PPARγ-mediated negative control of HIV replication in CD4^+^ T-cells, together with studies by other groups demonstrating similar effects on macrophages [64,66] and DC [113], a new generation of non-toxic PPARγ agonists may help reduce covert viral replication in HIV-infected subjects receiving ART. This additional therapeutic strategy may decrease the pool of HIV-permissive cells and thus subsequently reducing viral reservoirs especially when administered during the early phases of HIV infection.

## Methods

### Subjects

HIV-uninfected donors were recruited at the Montreal Chest Institute, McGill University Health Centre and Saint-Luc Hospital, Montréal, QC, Canada, through the FRSQ/SIDA-MI Network (Québec, Canada). Informed consent and Internal Review Board approval were obtained for all participants. Peripheral blood mononuclear cells (PBMC) (10^9^-10^10^ cells) were collected by leukapheresis as previously reported [123] and used frozen.

### Antibodies and polychromatic flow cytometry analysis

Fluorochrome-conjugated Abs used for polychromatic flow cytometry analysis were CD3-Pacific Blue (UCHT1), CD4-Alexa Fluor 700 (RPA-T4), CD45RA-APC-Cy7 (H1100), CCR4-PE-Cy7 (1G1), CXCR3-PE-Cy5 (1C6), CCR6-PE (11A9), CCR5-PE (2D7) and CEACAM1-FITC (B1.1) (BD Pharmingen), CD56-FITC (MEM188) (eBioscience), CD8-FITC (BW135/80), CD19-FITC (LT19), and MCAM-FITC (541-10B2) (Miltenyi Biotec). The viability dye Aqua Vivid (Invitrogen) was used to exclude dead cells from our analysis. Cells were analyzed by FACS using the BD LSRII cytometer, and BD Diva and FlowJo softwares (Tree Star, Inc). Positivity gates were placed using fluorescence minus one (FMO), as previously described [124].

### Magnetic (MACS) and fluorescence activated cell sorting (FACS)

Memory Th1Th17 and Th1 were sorted as previously described [31,125]. Briefly, total CD4^+^ T-cells were sorted from PBMCs by negative selection using magnetic beads (Miltenyi), memory (CD45RA-) Th1Th17 (CXCR3^+^CCR4^-^CCR6^+^ phenotype) and Th1 (CXCR3^+^CCR4^-^CCR6^-^ phenotype) subsets were sorted by FACS (BD Aria II cytometer) upon staining with anti-CD45RA-APC-Cy7, CCR4-PE-Cy7, CXCR3-PE-Cy5, CCR6-PE and a cocktail of FITC conjugated Abs to exclude CD8^+^ T-cells (CD8), NK cells (CD56), and B cells (CD19). The sorting gate was set on CD45RA^-^FITC^-^CCR4^-^CXCR3^+^ cells expressing or not CCR6. The purity of the cells was typically >98% for memory CD4^+^ T-cells (CD45RA^-^) and >95% for Th1Th17 and Th1 subsets (Additional file 1: Figure S1). The median frequency of memory Th1 and Th1Th17 cells within total CD4^+^ T-cells is 17.6% and 6.5%, respectively (n = 21) (data not shown). Typically, 4×10^6^ Th1Th17 cells can be isolated by MACS and FACS from approximately 10^8^ total CD4^+^ T-cells and 10^9^ PBMC (sorting yield >50%). For other experiments, memory CD4^+^ T-cells were sorted by negative selection using magnetic beads at a purity >98% as demonstrated by staining with CD3, CD4, and CD45RA Abs (data not shown).

### RNA isolation and microarray analysis

Sorted Th1Th17 and Th1 subsets were stimulated with immobilized CD3 and soluble CD28 Abs (1 μg/ml) for 3 days. Total RNA was isolated using the RNeasy kit (Qiagen) according to the manufacturer’s protocol and quantified by Pearl nanophotometer (Implen, Munich, Germany) (typically, 10^6^ cells yielded 3–5 μg RNA). Genome-wide analysis of gene expression was performed on total RNA extracted from Th1Th17 and Th1 cells of four different HIV-uninfected donors by Génome Québec (Montreal, Qc, Canada) using the Affymetrix technology. Briefly, the quality of total RNA was first tested using an Agilent 2100 Bioanalyzer chip. Then, high quality RNA was reverse transcribed and hybridized on the GeneChip® Human Genome U133 Plus 2.0 Array (Affymetrix). This chip includes 54,675 probe sets on a single array (*i.e.*, 47,000 transcripts and variants, including 38,500 well-characterized human genes). Gene expression data was analyzed using Bioconductor [126], an open-source software Library for the analyses of genomic data based on R, a programming language and environment for statistical computing and graphics (http://www.r-project.org). The R software package was used to preprocess and normalize the probes intensities using RMA [127] method for the four matched Th1Th17 and Th1 cell subsets.

### Microarray data analysis

Genes were filtered by detection call and by variance filters to allow a reduction in the number of tests and a corresponding increase in power of the differential gene expression analysis [128]. The resulting matrix showing 38,113 probes as present calls was log_2_ transformed and used as input for linear modeling using Bioconductor’s *limma* package (*Linear Models for Microarray Data*), which estimates the fold-change (FC) between predefined groups (Th1Th17 *vs.* Th1 cell subsets) by fitting a linear model and using an empirical Bayes method to moderate standard errors of the estimated log-fold changes for expression values from each gene. P-values from the resulting comparison were adjusted for multiple testing according to the method of Benjamini and Hochberg. This method controls the false discovery rate (FDR), which was set to 0.05. Determination of regulated gene expression is based on the nominal p-values. The entire microarray dataset and technical information requested by Minimum Information About a Microarray Experiment (MIAME) are available at the Gene Expression Omnibus (GEO) database under accession number GSE50175. Differentially expressed genes (cut-off 1.3-fold; p <0.05) were classified through Gene Ontology using the NetAffx web-based application (Affymetrix). Corresponding heat maps for biological function categories were generated using programming language R.

### Gene set enrichment analysis

Gene set enrichment analysis (GSEA) and *Molecular Signature DataBase* (MSigDB, Broad Institute, MIT, Cambridge, MA) were used to identify differentially expressed gene sets [41]. Microarray results were analyzed through collections C2, C3, and C5 of MsigDB latest version (4.0), yielding enrichment scores (ES) for each gene sets representing the difference between the observed and expected rankings from phenotype correlation. Merged to C2 are 28 gene sets collected from transcriptional analyses of the blood in immunological context named M x.x (eg. M 1.3) (their descriptions are not available through MsigDB, but can be found in Chaussabel et al. [129]), 24 immunological or signaling pathways from NetPath [130], and 132 gene sets from the Munich Information Center for Protein Sequences (CORUM MIPS; [131]). ES was then estimated by using a pre-ranked genes procedure based on the t-statistics of every transcript used in the linear model for gene expression. Nominal p-values were adjusted for multiple testing using the Benjamini and Hochberg method to control the FDR. Normalized enrichment scores (NES) were calculated for each gene set to account for the size of the set.

### Quantitative SYBR Green real- time RT-PCR

Total RNA was isolated using RNeasy kit (Qiagen) from Th1Th17 and Th1 subsets of HIV-uninfected individuals. The quality (260/280 ratio) and quantity of RNA collected were measured by a Pearl nanophotometer (Implen, Munich, Germany). One step SYBR Green real-time RT-PCR (Qiagen) was carried out in a LightCycler 480 II (Roche) according to manufacturer’s recommendations. CXCR6, PPARγ, SERPINB6, PTK2, PTPN13, MAP3K4, ARNTL, and CTSH primers were purchased from Qiagen (QuantiTect Primer Assay). Quantifications were performed as previously described [95]. Briefly, 5 ng total RNA was reverse transcribed in 20 μl SYBR Green mix (Qiagen) containing 0.5 μM primers. Agarose gel electrophoresis was used to visualize the size of the amplification products and to perform cDNA purification using the QIAquick Gel Extraction Kit (Qiagen) for standard curve preparation. Serial dilution of purified RT-PCR products (20,000; 2,000; 200; 20; 2; 0.2 fg cDNA) was used for the absolute quantification of target gene expression. The expression of each gene was normalized relative to the internal control 28S rRNA levels (forward 5′-CGAGATTCCTGTCCCCACTA-3′; reverse 5′-GGGGCCACCTCCTTATTCTA-3′, IDT, [95]). Melting curve analysis performed after real-time amplification revealed the uniformity of thermal dissociation profile per amplification product. Samples without template or without reverse transcriptase were used as negative controls. Each RT-PCR reaction was performed in triplicata.

### Fluorescent microscopy and quantitative image analysis

Sorted Th1Th17 and Th1 cells were stimulated *via* CD3/CD28 for 3 days (1 μg/ml). Cells were harvested and placed into poly-L-lysine-coated 8-wells glass culture slides (BD Biosciences) (10^5^ cells/well), spun down for 1 min at 1500 rpm, and fixed with 4% formaldehyde for 30 min at room temperature. Cells were permeabilized 5 min at room temperature in PBS, 1% BSA, 0.5% Triton X-100. Cells were stained with rabbit anti-human PPARγ Abs (C26H12, Cell Signaling) overnight at 4°C and then with Alexa Fluor 488-conjugated goat anti-rabbit Abs (Invitrogen) for 2 h at room temperature. Slides were mounted using ProLong Gold Antifade medium with the nuclear dye DAPI (Invitrogen, Molecular Probes). Epi-fluorescent and Spinning Disc confocal microscopy images were carried out on an automated Cell Observer Z1® microscope (Carl Zeiss) using the AxioVision 4.8.2 software (Carl Zeiss), except otherwise mentioned in the figures. For statistical analysis of protein expression in Th1Th17 and Th1 cells, random epi-fluorescent images were acquired with the 40x oil immersion objective (numerical aperture, NA, 1.3). All acquisitions between the different T-cell subsets were performed with the same illumination status in the same run. Integrated density was measured after background subtraction with ImageJ software (NIH). Data were compared by analysis of integrated density/area for 50–100 cells/subset. For the analysis of PPARγ cellular localization in different subsets, spinning disc confocal images were acquired using the 100x oil immersion objective (NA, 1.46) and maximum intensity projection of 0.2 μm z-stack sections were realized using ImageJ software after background subtraction. The ratio between nuclear and total PPARγ expression (integrated density units) was calculated on 10–16 sections/cell and mean±SD were calculated on five different cells representative/subset.

### HIV infection and quantification of viral replication

The following HIV-1 molecular clones were used in this study: ***(i)*** the replication competent wild type CCR5-using (R5) NL4.3 strain with the BAL envelope (NL4.3BAL wt); ***(ii)*** the NL4.3BAL expressing *gfp* in place of *nef* (NL4.3BAL-GFP); and ***(iii)*** single round env-deficient NL4.3 provirus expressing *gfp* in place of *nef* pseudotyped with the VSV-G envelope (HIV-VSVG-GFP) that enter cells independently of HIV receptor and coreceptors, as previously reported [38]. The HIV stocks were produced and titrated as previously described [31] and used to infect cells (10 or 50 ng HIV-p24 *per* 10^6^ cells). Unbound HIV was removed by extensive washing, and cells were cultured at a concentration of 10^6^ cells per milliliter in RPMI1640, 10% FBS, and IL-2 (5 ng/ml; R&D Systems). Cell culture supernatants were harvested every 3–4 days and viral replication was measured by HIV-p24 ELISA. In parallel, cells were harvested at day 3 post-infection and cell lysates were used for real-time PCR quantification of integrated HIV-DNA (10^5^ cells *per* test in triplicata), as previously described [6,31].

### PPARγ RNA interference

PBMC were thawed and rested overnight at 37°C. Memory CD4^+^ T-cells were sorted by negative selection using magnetic beads (Miltenyi Biotec). Cells were stimulated *via* CD3/CD28 (immobilized anti-CD3 and soluble anti-CD28 Abs, NA/LE, 1 μg/ml, BD Pharmingen) in RPMI1640, 10% FBS, 1% penicillin/streptomycin (PS). At day 2, cells were harvested, washed in warm RPMI1640 w/o FBS and PS and nuclofected with 100 μM PPARγ-specific or non-targeting (NT1) siRNA (ON-TARGETplus SMART pool, Dharmacon) using the Amaxa Human T cell Nucleofector Kit (Amaxa, Lonza) according to the manufacturer’s protocol. Briefly, cells were suspended in supplemented NF solution (100 μl/2x10^6^ cells) and nucleofected using the Amaxa Nucleofector II Device and the human activated T-cell protocol (T-20). Cells (2×10^6^ cells) were immediately transferred into 48-well plates containing 1 ml of warm RPMI1640, 10% FBS, 5 ng/ml IL-2, w/o antibiotics and cultured for 24 h at 37°C. Cells were washed and exposed to replication competent HIV (NL4.3BaL, 10 ng/10^6^ cells) or to single round VSV-G pseudotyped HIV (HIV-VSVG-GFP) strains as described above. Cells were washed and cultured up to 6 days. At 24 h, 48 h and 72 h post-nucleofection, the efficiency of RNA silencing was assessed at the level of mRNA (SYBR Green real-time RT-PCR) and protein (fluorescence microscopy analysis).

### Cell viability and proliferation

Magnetic bead-sorted memory CD4^+^ T-cells were stimulated with CD3/CD28 Abs for 2 days and then nuclofected with 100 μM PPARγ-specific or non-targeting (NT1) siRNA as described above. Cells were cultured in RPMI1640, 10% FBS, 5 ng/ml IL-2, w/o antibiotics for 24 h at 37°C. Cells were washed and cultured for additional 5 days. Cells were stained with the LIVE/DEAD® Fixable Dead Cell Stain Kit (Aqua Vivid, Invitrogen) to exclude dead cells from analysis. Intracellular staining using Ki-67 Abs was performed to quantify proliferation of nuclofected cells. Samples were analyzed by flow cytometry. Absolute cell numbers were determined using the FlowCount fluorospheres (BeckmanCoulter).

### Statistics

All statistical analyses were performed using the GraphPad Prism 5 software and details are included in Figure legends.

## Competing interests

The authors declare that they have no competing interests.

## Authors’ contributions

AB, ACB, and YZ performed the majority of experiments and contributed to figure design and manuscript writing. JPG performed statistical analysis of microarray data. PM sorted the cells and extracted the RNA for microarrays and contributed to the validation of results by RT-PCR. AG and SDF performed real-time PCR analysis. VSW contributed to cell sorting. MAJ, JPR and CT were involved in sample collection and experimental design. PA designed the study, designed the figures, and wrote the manuscript. All authors read and approved the manuscript.

## Supplementary Material

Additional file 1: Figure S1 Flow cytometry sorting of Th1Th17 and Th1 cells. Total CD4^+^ T-cells were sorted from PBMCs by negative selection using magnetic beads (Miltenyi). Cells were labeled with a cocktail of CD45RA, CD8, CD19, CD56, CCR4, CXCR3 and CCR6 Abs in view of flow cytometry sorting. **(A)** Cells were selected based on their size (FSC height and wide) and granularity (SSC height and wide) to exclude debris and aggregates (P3). Memory CD4^+^ T-cells were then identified by their CD45RA- phenotype (P4) while excluding possible contamination by CD8^+^ T cells, NK cells or B lymphocytes using the expression of CD8, CD56 and CD19, respectively, in the same color channel (Lineage). Memory CD4^+^ T cells were separated by their expression of CXCR3 but not CCR4 (P5), and the expression of CCR6 identified CCR4^-^CXCR3^+^CCR6^+^ (Th1Th17 polarization profile) and CCR4^-^CXCR3^+^CCR6^-^ (Th1 polarization profile). Shown values correspond to the percentages of events included in the selection window relative to single cells (P3). **(B)** A sample of each of the Th1Th17 and Th1 subsets was collected after cell sorting and analyzed by flow cytometry to obtain quality controls (QC). Click here for file

Additional file 2: Table S1 Probe sets upregulated in Th1Th17 *vs.* Th1 cells. Sorted Th1Th17 and Th1 cell subsets from four HIV-uninfected donors were stimulated *via* CD3/CD28 for 3 days. Total RNA was extracted and reverse transcribed into cDNA to be hybridized on Human U133 Plus 2.0 including >47,000 probe sets per chip (Affymetrix). One-way ANOVA analysis was performed to identify differentially expressed genes (p-value <0.05). Shown is the list of the 438 probe sets with a preferential expression in Th1Th17 *vs.* Th1, corresponding to 392 known genes and 46 unknown transcripts. Click here for file

Additional file 3: Table S2 Probe sets downregulated in Th1Th17 *vs.* Th1 cells. Sorted Th1Th17 and Th1 cell subsets from four HIV-uninfected donors were stimulated *via* CD3/CD28 for 3 days. Total RNA was extracted and reverse transcribed into cDNA to be hybridized on Human U133 Plus 2.0 including >47,000 probe sets per chip (Affymetrix). One-way ANOVA analysis was performed to identify differentially expressed genes (p-value <0.05). Shown is the list of the 342 probe sets with a preferential expression in Th1 *vs.* Th1Th17, corresponding to 268 known genes and 74 unknown transcripts. Click here for file

Additional file 4: Figure S2 *Gene Set Enrichment Analysis* (GSEA) of differentially expressed in Th1Th17 *vs.* Th1 cells. Expression levels of the 38,113 probe sets obtained by Affymetrix were used to search the C2 Canonical pathways **(A)**, C3 Transcription factors and miRNA **(B)** and C5 Gene Ontology **(C)** collections of the *Molecular Signature DataBase* (MsigDB, Broad Institute) through a GSEA. The GSEA identified several biological functions and signaling pathways differentially expressed in Th1Th17 *vs.* Th1 cells (FDR-value <0.05). Shown are pathways, transcription factors, and gene sets from Gene Ontology selected based on their normalized enrichment score (NES > 1.5). Click here for file

Additional file 5: Figure S3 Preferential expression of the adhesion molecules CEACAM1 and MCAM on Th1Th17 *vs.* Th1 cells *ex vivo*. PBMC were stained with a cocktail of fluorochrome-labeled CD3, CD4, CD45RA, CCR4, CXCR3, CCR6, and CEACAM1 or MCAM Abs and analyzed by polychromatic flow cytometry. A viability staining (Vivid) was used to exclude dead cells. **(A)** Shown is the gating strategy for the identification of memory CD3^+^CD4^+^CD45RA^-^ T-cells with a Th1Th17 (CCR6^+^CCR4^-^CXCR3^+^) and Th1 (CCR6^-^CCR4^-^CXCR3^+^) phenotype. Shown is expression of CEACAM1 (**B**-**C**) and MCAM (**D**-**E**) on Th1Th17 *vs.* Th1 cells. Results in **A, B,** and **D** are from one donor representative of results generated with cells from seven different donors. Results in **C** and **E** are statistical analysis of CEACAM1 and MCAM expression in seven different HIV-uninfected individuals. Paired *t*-Test p-values are indicated in the figures. (PDF 36 kb) Click here for file

Additional file 6: Figure S4 Confocal microscopy quantification of PPARγ protein knock down upon RNA interference. CD4^+^ T-cells were isolated, activated *via* TCR and nucleofected with PPARγ *vs.* NT1 siRNA as described in Figure 6. At 24 h, 48 h and 72 h post-nucleofection, cells were fixed on slides and stained with PPARγ Abs as in Figure 5. **(A)** PPARγ expression was observed using fluorescence microscopy and 40x immersion oil objective (NA, 1.3). Shown are statistical analysis of PPARγ expression at 24 h, 48 h and 72 h post-nucleofection in n = 3 different donors (n > 100 cells *per* subsets *per* donor). Horizontal red lines indicate median values. Unpaired *p*-values are indicated on the figures. **(B)** Shown is PPARγ expression in cells 72 h after PPARγ silencing from one donor representative of n = 3 donors. Images are maximum intensity z-projection of z-stack from one representative field *per* experimental condition observed with a 63x oil objective (NA, 1.46) in a LSM780 microscope (Zeiss) with an additional 2.6 numerical zoom. Click here for file

Additional file 7: Figure S5 Kinetics of PPARγ mRNA expression upon RNA interference. CD4^+^ T-cells were isolated, activated *via* TCR and nucleofected with PPARγ *vs.* NT1 siRNA as described in Figure 6. Total RNA was extracted from cells at 24 h, 48 h, and 72 h post-nucleofection. Levels of PPARγ mRNA were quantified by real-time RT-PCR in n = 3 different subjects relative to the internal control 28S rRNA. PPARγ expression in NT1 siRNA nucleofected cells was considered 100%. Click here for file

Additional file 8: Figure S6 Effects of PPARγ agonists on cell viability, proliferation and CD4 and CCR5 expression. Memory CD4^+^ T-cells from 3 healthy donors were isolated by negative selection using magnetic beads (Miltenyi), stained with CFSE (0.5 μM), and stimulated *via* CD3/CD28 for 3 days. Cells were then cultured for three additional days with IL-2 (5 ng/ml) in the presence or absence of different concentrations of the PPARγ agonists RGZ (1, 10, 50, 100 μM) and PGJ2 (1, 5, 10, 20, 50 μM). Cells were stained with the viability dye Vivid and with CD4 and CCR5 Abs and were analyzed by polychromatic flow cytometry. **(A)** Shown is the gating strategy for the identification of viable cells (Vivid^-^), CFSE^low^ proliferating cells, and cells expressing CD4 and CCR5, when T-cells were cultured in the absence of PPARγ agonists. Depicted are results from one donor representative of results obtained with three different donors. Shown are changes induced by PPARγ agonists in cell viability **(B)**, cell proliferation **(C)**, and the expression of CD4 and CCR5 **(D-E)**. Depicted are the % (left panels) and the *mean fluorescence intensity* (MFI) (right panels) of CFSE, CD4, and CCR5 expression. Results in **B-E** were generated with cells from three different donors. Horizontal lines represent median values. Click here for file

Additional file 9: Figure S7 Rosiglitazone triggers the nuclear translocation of PPARγ. Memory CD4^+^ T-cells were stimulated *via* CD3/CD28 for 3 days and cultured in the presence or absence of the PPARγ agonist RGZ (50 μM) and/or the antagonist T007907 (10 μM) for 20 h at 37°C. Cells were seeded on poly-L-lysine-coated 8-well glass culture slides and stained intracellularly for PPARγ, as described in Material and Methods. Slides were mounted with the *ProLong Gold Antifade* reagent containing the nuclear dye DAPI. Slides were observed by confocal microscopy (Carl Zeiss). **(A)** Shown are images taken using the AxioVision 4.8.2 software at 100x oil magnification and z-stack reconstruction of 0.2 μm virtual sections. **(B)** Shown are statistical analysis of the nuclear *vs.* cytoplasm ratio of PPARγ expression in cells treated or not with RGZ and/or T007907 performed with the ImageJ software (NIH) on n = 15 cells per condition. Unpaired *t*-Test p-values are indicated on the graph. Results in **A-B** are from one donor, representative of results observed with cells from four different donors. Click here for file
