# Supplementary figures and images for: Transcriptional profiling reveals molecular signatures associated with HIV permissiveness in Th1Th17 cells and identifies Peroxisome Proliferator-Activated Receptor Gamma as an intrinsic negative regulator of viral replication
Source: Retrovirology. 2013 Dec 21;10:160. doi: 10.1186/1742-4690-10-160 (PMC3898812; doi:10.1186/1742-4690-10-160)

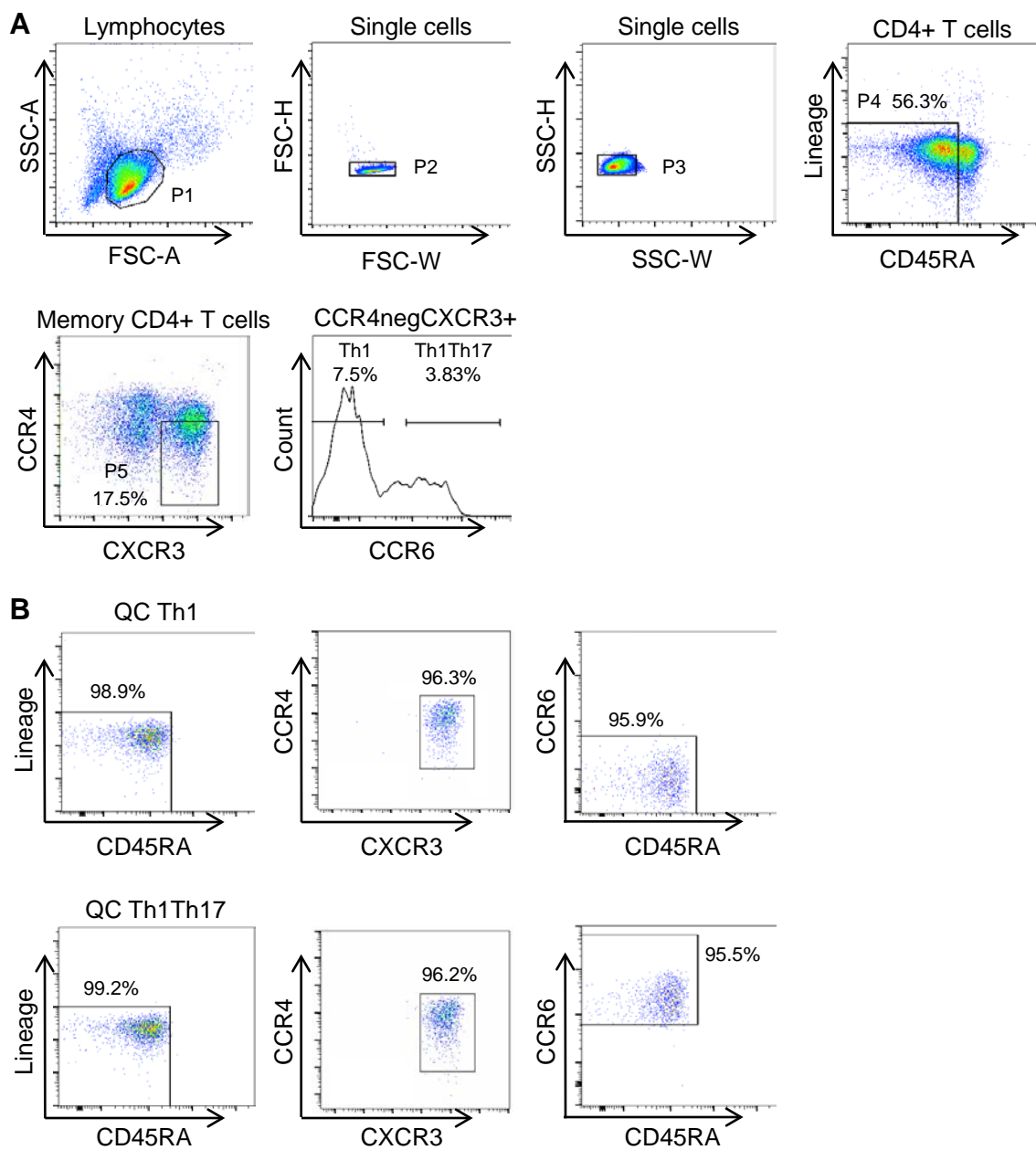

Supplement: Additional file 1: Figure S1 — Flow cytometry sorting of Th1Th17 and Th1 cells. Total CD4+ T-cells were sorted from PBMCs by negative selection using magnetic beads (Miltenyi). Cells were labeled with a cocktail of CD45RA, CD8, CD19, CD56, CCR4, CXCR3 and CCR6 Abs in view of flow cytometry sorting. (A) Cells were selected based on their size (FSC height and wide) and granularity (SSC height and wide) to exclude debris and aggregates (P3). Memory CD4+ T-cells were then identified by their CD45RA- phenotype (P4) while excluding possible contamination by CD8+ T cells, NK cells or B lymphocytes using the expression of CD8, CD56 and CD19, respectively, in the same color channel (Lineage). Memory CD4+ T cells were separated by their expression of CXCR3 but not CCR4 (P5), and the expression of CCR6 identified CCR4-CXCR3+CCR6+ (Th1Th17 polarization profile) and CCR4-CXCR3+CCR6- (Th1 polarization profile). Shown values correspond to the percentages of events included in the selection window relative to single cells (P3). (B) A sample of each of the Th1Th17 and Th1 subsets was collected after cell sorting and analyzed by flow cytometry to obtain quality controls (QC). [file 1742-4690-10-160-S1.pdf]

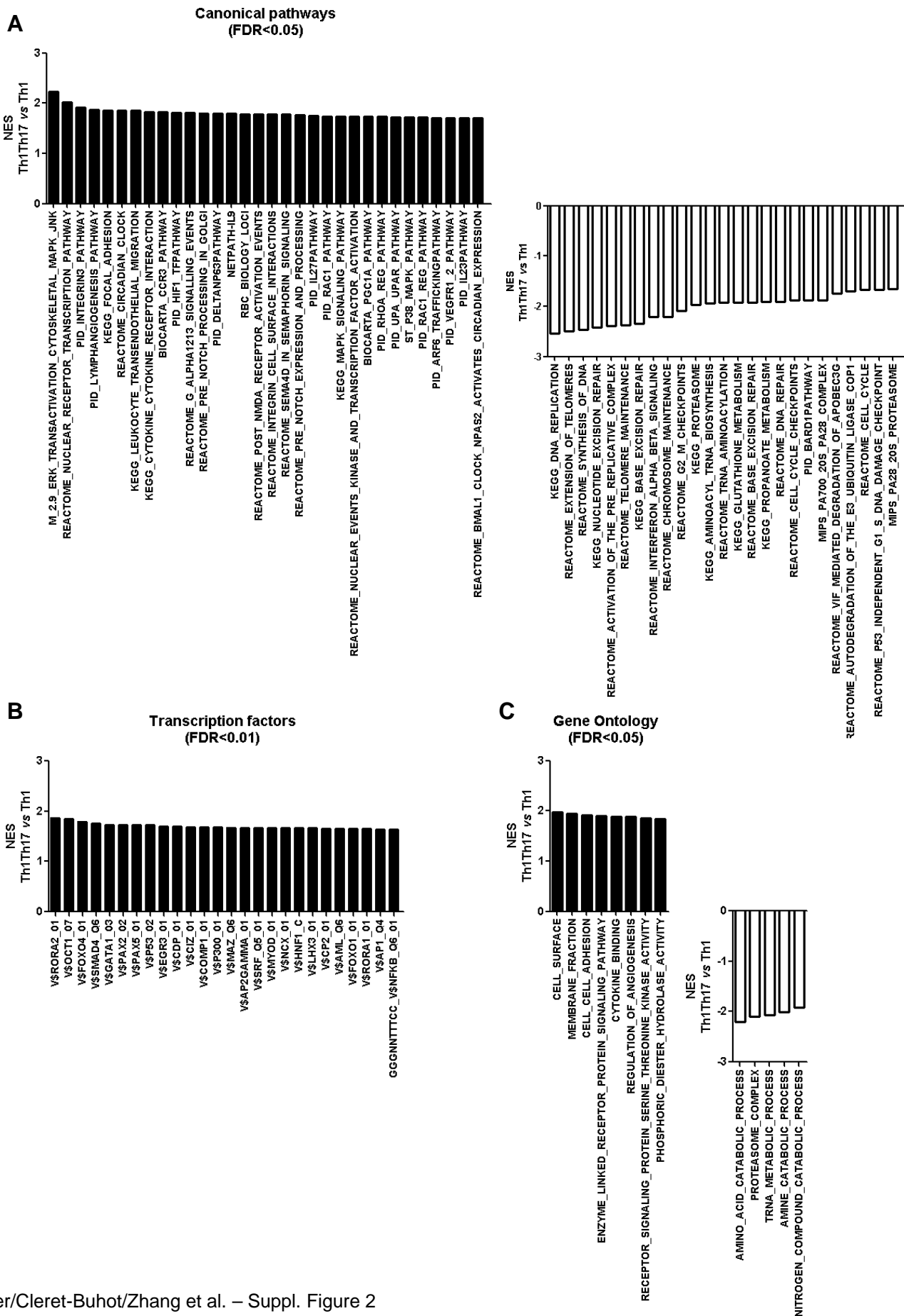

Supplement: Additional file 4: Figure S2 — Gene Set Enrichment Analysis (GSEA) of differentially expressed in Th1Th17 vs. Th1 cells. Expression levels of the 38,113 probe sets obtained by Affymetrix were used to search the C2 Canonical pathways (A), C3 Transcription factors and miRNA (B) and C5 Gene Ontology (C) collections of the Molecular Signature DataBase (MsigDB, Broad Institute) through a GSEA. The GSEA identified several biological functions and signaling pathways differentially expressed in Th1Th17 vs. Th1 cells (FDR-value <0.05). Shown are pathways, transcription factors, and gene sets from Gene Ontology selected based on their normalized enrichment score (NES > 1.5). [file 1742-4690-10-160-S4.pdf]

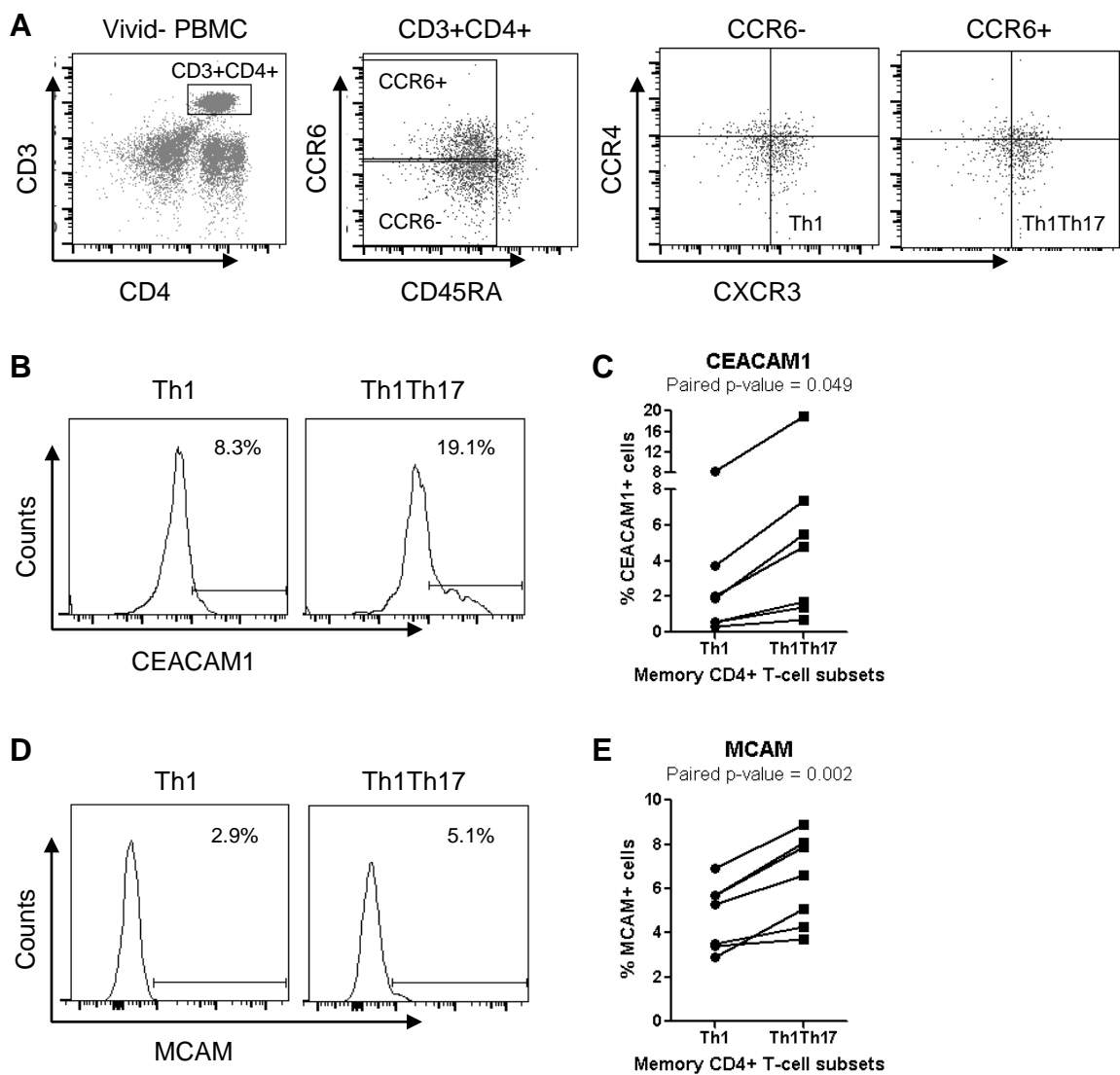

Supplement: Additional file 5: Figure S3 — Preferential expression of the adhesion molecules CEACAM1 and MCAM on Th1Th17 vs. Th1 cells ex vivo. PBMC were stained with a cocktail of fluorochrome-labeled CD3, CD4, CD45RA, CCR4, CXCR3, CCR6, and CEACAM1 or MCAM Abs and analyzed by polychromatic flow cytometry. A viability staining (Vivid) was used to exclude dead cells. (A) Shown is the gating strategy for the identification of memory CD3+CD4+CD45RA- T-cells with a Th1Th17 (CCR6+CCR4-CXCR3+) and Th1 (CCR6-CCR4-CXCR3+) phenotype. Shown is expression of CEACAM1 (B-C) and MCAM (D-E) on Th1Th17 vs. Th1 cells. Results in A, B, and D are from one donor representative of results generated with cells from seven different donors. Results in C and E are statistical analysis of CEACAM1 and MCAM expression in seven different HIV-uninfected individuals. Paired t-Test p-values are indicated in the figures. (PDF 36 kb) [file 1742-4690-10-160-S5.pdf]

**A**

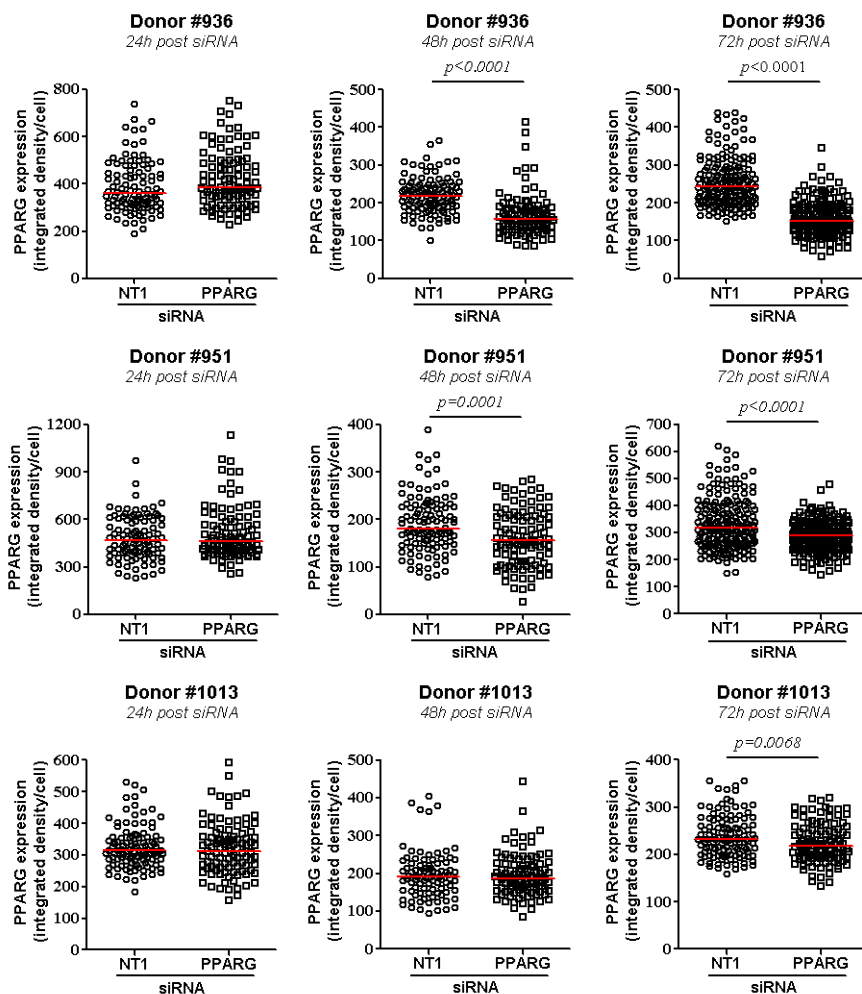

**B**

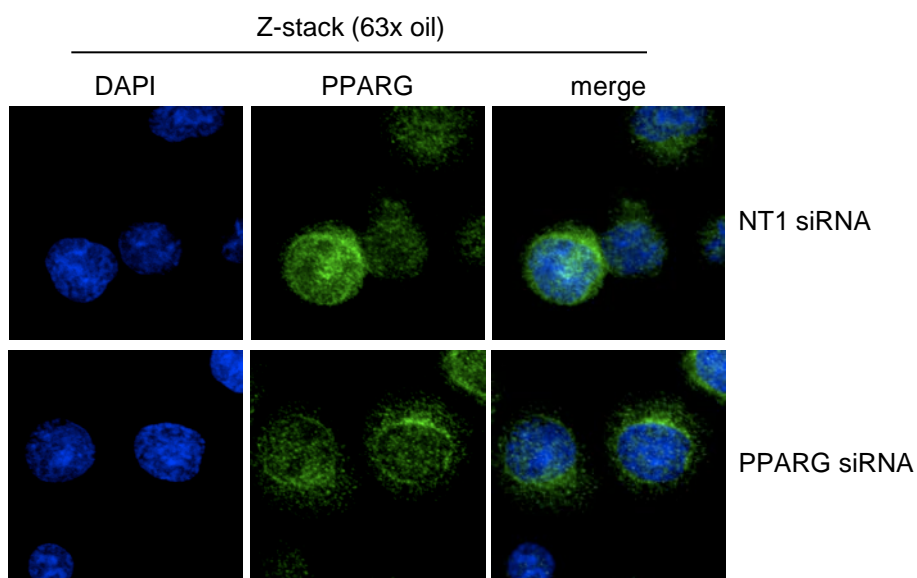

Supplement: Additional file 6: Figure S4 — Confocal microscopy quantification of PPARγ protein knock down upon RNA interference. CD4+ T-cells were isolated, activated via TCR and nucleofected with PPARγ vs. NT1 siRNA as described in Figure 6. At 24 h, 48 h and 72 h post-nucleofection, cells were fixed on slides and stained with PPARγ Abs as in Figure 5. (A) PPARγ expression was observed using fluorescence microscopy and 40x immersion oil objective (NA, 1.3). Shown are statistical analysis of PPARγ expression at 24 h, 48 h and 72 h post-nucleofection in n = 3 different donors (n > 100 cells per subsets per donor). Horizontal red lines indicate median values. Unpaired p-values are indicated on the figures. (B) Shown is PPARγ expression in cells 72 h after PPARγ silencing from one donor representative of n = 3 donors. Images are maximum intensity z-projection of z-stack from one representative field per experimental condition observed with a 63x oil objective (NA, 1.46) in a LSM780 microscope (Zeiss) with an additional 2.6 numerical zoom. [file 1742-4690-10-160-S6.pdf]

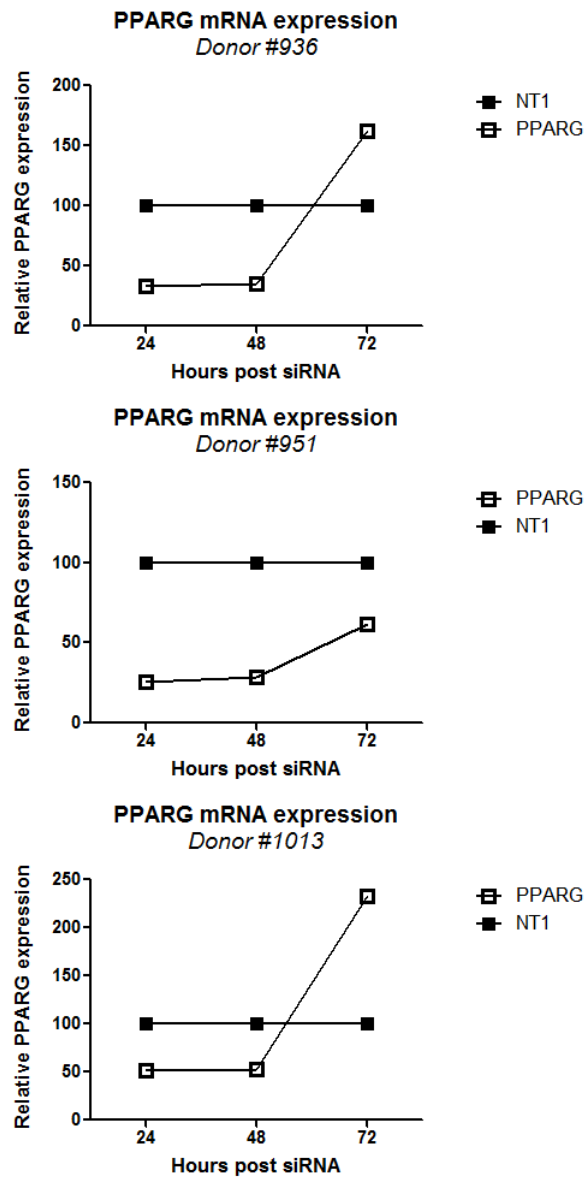

Supplement: Additional file 7: Figure S5 — Kinetics of PPARγ mRNA expression upon RNA interference. CD4+ T-cells were isolated, activated via TCR and nucleofected with PPARγ vs. NT1 siRNA as described in Figure 6. Total RNA was extracted from cells at 24 h, 48 h, and 72 h post-nucleofection. Levels of PPARγ mRNA were quantified by real-time RT-PCR in n = 3 different subjects relative to the internal control 28S rRNA. PPARγ expression in NT1 siRNA nucleofected cells was considered 100%. [file 1742-4690-10-160-S7.pdf]

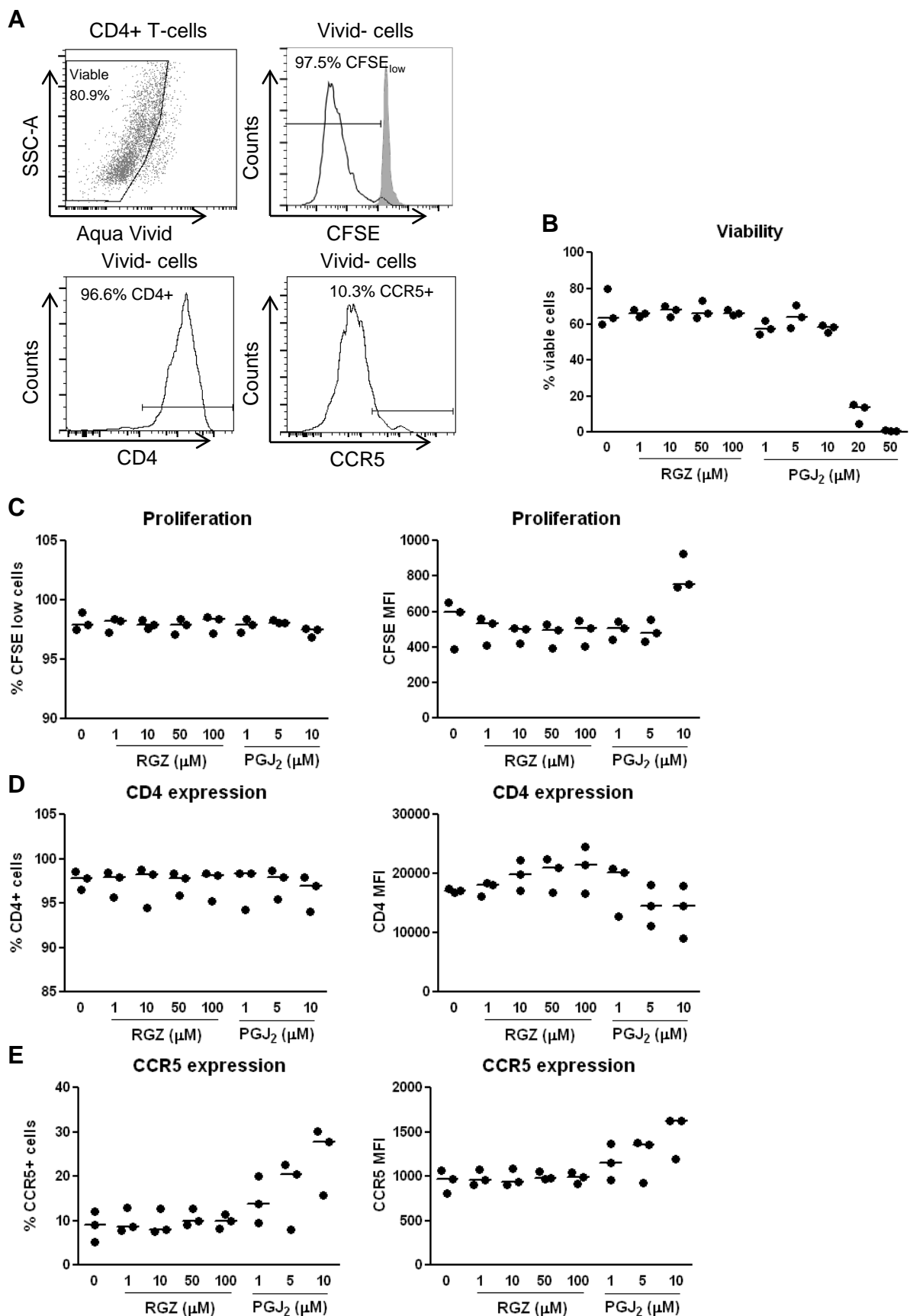

Supplement: Additional file 8: Figure S6 — Effects of PPARγ agonists on cell viability, proliferation and CD4 and CCR5 expression. Memory CD4+ T-cells from 3 healthy donors were isolated by negative selection using magnetic beads (Miltenyi), stained with CFSE (0.5 μM), and stimulated via CD3/CD28 for 3 days. Cells were then cultured for three additional days with IL-2 (5 ng/ml) in the presence or absence of different concentrations of the PPARγ agonists RGZ (1, 10, 50, 100 μM) and PGJ2 (1, 5, 10, 20, 50 μM). Cells were stained with the viability dye Vivid and with CD4 and CCR5 Abs and were analyzed by polychromatic flow cytometry. (A) Shown is the gating strategy for the identification of viable cells (Vivid-), CFSElow proliferating cells, and cells expressing CD4 and CCR5, when T-cells were cultured in the absence of PPARγ agonists. Depicted are results from one donor representative of results obtained with three different donors. Shown are changes induced by PPARγ agonists in cell viability (B), cell proliferation (C), and the expression of CD4 and CCR5 (D-E). Depicted are the % (left panels) and the mean fluorescence intensity (MFI) (right panels) of CFSE, CD4, and CCR5 expression. Results in B-E were generated with cells from three different donors. Horizontal lines represent median values. [file 1742-4690-10-160-S8.pdf]

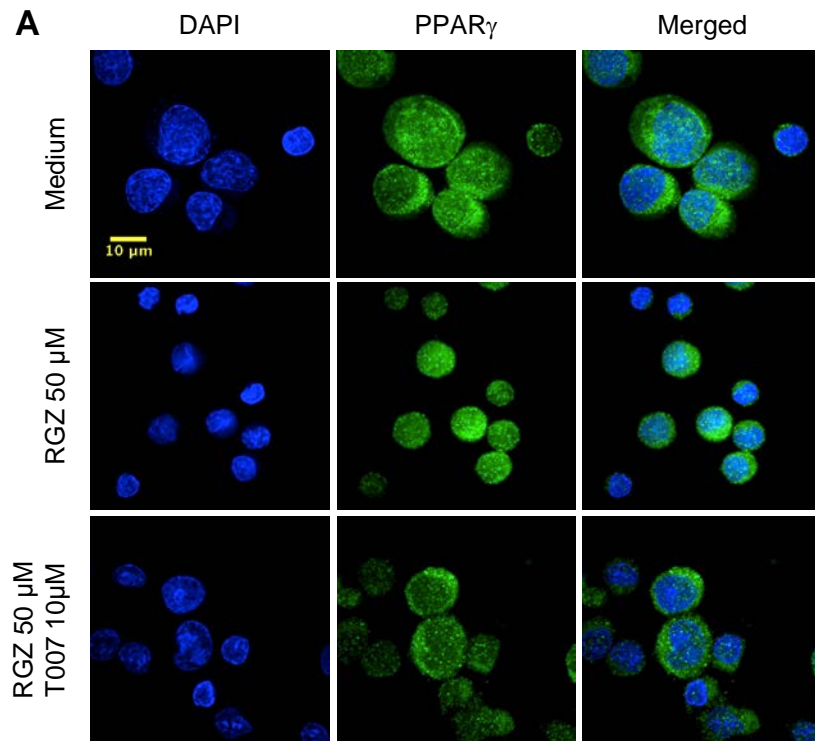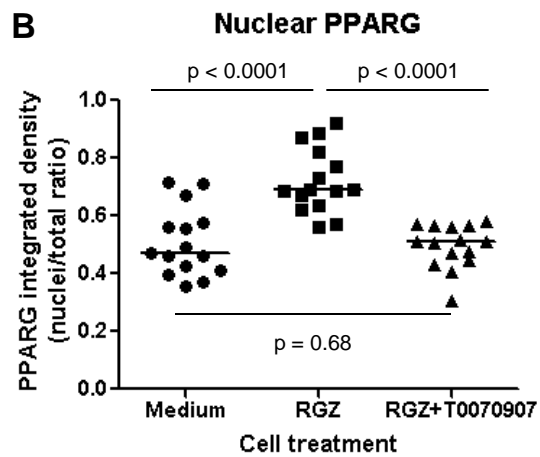

Supplement: Additional file 9: Figure S7 — Rosiglitazone triggers the nuclear translocation of PPARγ. Memory CD4+ T-cells were stimulated via CD3/CD28 for 3 days and cultured in the presence or absence of the PPARγ agonist RGZ (50 μM) and/or the antagonist T007907 (10 μM) for 20 h at 37°C. Cells were seeded on poly-L-lysine-coated 8-well glass culture slides and stained intracellularly for PPARγ, as described in Material and Methods. Slides were mounted with the ProLong Gold Antifade reagent containing the nuclear dye DAPI. Slides were observed by confocal microscopy (Carl Zeiss). (A) Shown are images taken using the AxioVision 4.8.2 software at 100x oil magnification and z-stack reconstruction of 0.2 μm virtual sections. (B) Shown are statistical analysis of the nuclear vs. cytoplasm ratio of PPARγ expression in cells treated or not with RGZ and/or T007907 performed with the ImageJ software (NIH) on n = 15 cells per condition. Unpaired t-Test p-values are indicated on the graph. Results in A-B are from one donor, representative of results observed with cells from four different donors. [file 1742-4690-10-160-S9.pdf]
